# Trends in Cardiovascular Risk Factors in Canada: Variation by Migration and Temporal Factors, 2001-2018

**DOI:** 10.1016/j.cjco.2024.04.006

**Published:** 2024-04-25

**Authors:** Kitty Y.A. Chen, Anan Bader Eddeen, Carol Bennett, Warsame Yusuf, Deirdre Hennessy, Joel D. Barnes, Douglas G. Manuel

**Affiliations:** a Clinical Epidemiology Program, Ottawa Hospital Research Institute, Ottawa, Ontario, Canada; b Dalla Lana School of Public Health, University of Toronto, Toronto, Ontario, Canada; c Institute for Clinical Evaluative Sciences, Ottawa, Ontario, Canada; d National Microbiology Laboratory, Public Health Agency of Canada, Guelph, Ontario, Canada; e Health Analysis, Statistics Canada, Ottawa, Ontario, Canada; f Department of Family Medicine, University of Ottawa, Ottawa, Ontario, Canada

## Abstract

**Background:**

Cardiovascular disease is a leading cause of death in Canada, but how the major cardiovascular risk factors vary across ethnicity and immigration status has yet to be examined.

**Methods:**

Using data from the Canadian Community Health Surveys, national trends in health conditions (hypertension, diabetes, high blood cholesterol level, and obesity) and health behaviours (smoking, activity levels, and alcohol consumption) were estimated for the period 2001-2018. In this cross-sectional study, the trends were then compared across sex, age, ethnicity, and immigration status.

**Results:**

A total of 1,065,391 respondents were examined, for the period 2001-2018. During the study period, the prevalence of the following risk factors increased in Canada over time, as follows: diabetes by 54.5%; hypertension by 23.4%; and obesity by 32.3%. For health behaviours, smoking prevalence decreased overall, especially in racialized populations. Heavy drinking was most prevalent for nonracialized and non-Indigenous Canadian-born populations, and was of lowest prevalence among racialized immigrants. Physical inactivity was most prevalent for racialized immigrant populations. The prevalence of self-reported heart disease decreased by 21.0%, except for racialized established immigrants (≥ 10 years since immigration to Canada), who had a 4.2% increase.

**Conclusions:**

During this study period, decreases occurred in the prevalences of smoking and physical inactivity, along with increases in obesity, diabetes, and hypertension prevalences. By migration-group status, established immigrants in Canada had a higher prevalence of cardiovascular disease risk factors compared to that among their Canadian-born counterparts. Migration gaps should be considered in future interventions targeted at reducing these cardiovascular risk factors in Canada.

## Résumé

**Contexte:**

Les maladies cardiovasculaires sont une cause majeure de décès au Canada, mais la manière dont les principaux facteurs de risque cardiovasculaire varient en fonction de l’origine ethnique et du statut d’immigration n’a encore jamais été évaluée.

**Méthodologie:**

Des tendances nationales dans certains problèmes de santé (hypertension, diabète, hypercholestérolémie et obésité) et certains comportements liés à la santé (tabagisme, niveaux d’activité et consommation d’alcool) ont été dégagées à partir des données de l’Enquête sur la santé dans les collectivités canadiennes pour la période de 2001 à 2018. Ces tendances ont ensuite été comparées en fonction du sexe, de l’âge, de l’origine ethnique et du statut d’immigration dans le cadre de la présente étude transversale.

**Résultats:**

Au total, 1 065 391 répondants ont été examinés pour la période de 2001 à 2018. Durant la période de l’étude, la prévalence des facteurs de risque suivants a graduellement augmenté au Canada : diabète, de 54,5 %; hypertension, de 23,4 %; obésité, de 32,3 %. Dans le cas des comportements liés à la santé, la prévalence du tabagisme a globalement diminué, surtout dans les populations racialisées. La consommation excessive d’alcool était plus fréquente dans les populations non racialisées et non autochtones d’origine canadienne, et moins fréquente chez les immigrants racialisés. L’inactivité physique était particulièrement répandue dans les populations immigrantes racialisées. La prévalence des maladies cardiaques auto-déclarées a diminué de 21,0 %, sauf chez les immigrants établis racialisés (≥ 10 ans depuis l’arrivée au Canada), qui a connu une hausse de 4,2 %.

**Conclusions:**

Durant la période de l’étude, la prévalence du tabagisme et de l’inactivité physique a diminué tandis que celle de l’obésité, du diabète et de l’hypertension a augmenté. D’après le statut du groupe de migration, la prévalence des facteurs de risque de maladies cardiovasculaires était plus élevée chez les immigrants établis au Canada que chez leurs homologues nés au Canada. Il convient de prendre en considération les différences liées à la migration dans les interventions futures visant à réduire ces facteurs de risque cardiovasculaire au Canada.

Cardiovascular disease (CVD) is the leading cause of death in Canada and globally.^1^ Management of influential risk factors, such as smoking, followed by high blood pressure, physical inactivity, obesity, diabetes, and poor diet, have been shown to reduce the incidence of cardiovascular events and mortality.^2^ Although previous studies have examined the prevalence of cardiovascular risk factors in Canada over shorter time frames, no studies have explored these prevalences comprehensively over a longer and more recent timespan, while incorporating additional risk factors, such as physical inactivity, alcohol consumption, and high blood cholesterol level.^3^, ^4^, ^5^ Sustained efforts are required to decrease the prevalence of cardiovascular risk factors further.

Despite the overall improvement in CVD outcomes in the past decade, noticeable differences in CVD risk continue to persist among various racial and ethnic groups.^6^ Although racial and ethnic disparities have been reported extensively, how this varies by cardiovascular risk factors in Canada has yet to be evaluated over an extensive period of time. Currently, immigrants comprise more than 20% of Canada’s population, and the proportion continues to increase.^7^ Considering all immigrants as a uniform group blurs distinctions in health outcomes that stem from the wide range of ethnicities within this population.^8^ With the growing diversity in Canada, a greater understanding of the dynamic nature of socioeconomic determinants of cardiovascular health between the migrant population and the host population not only can help mitigate the unequal burden of CVD, but also can guide contextually appropriate health interventions for subpopulations. Given this context, the purpose of this study was to examine national trends in heart disease and a wide range of related risk factors, such as ethnicity and immigration status, among the Canadian population, including key subgroups, spanning the period from 2001 to 2018.

## Methods

### Study data

The Canadian Community Health Survey (CCHS) is a cross-sectional survey conducted by Statistics Canada, gathering information on health status, health determinants, and sociodemographic characteristics of individuals aged ≥ 12 years in Canada. The survey covers the 10 provinces and 3 territories, excluding persons living on First Nations reserves and settlements, full-time members of the Canadian Forces, and institutionalized persons. Data were collected through computer-assisted personal and telephone interview software in English and French. Each Statistics Canada regional office recruited interviewers with various language competencies, as a means to remove language barriers. Aspects of the census, such as the sampling unit and subsampling, have been incorporated into the CCHS to allow for stratification of population characteristics. The CCHS has undergone periodic updates, and a major redesign occurred in 2015, which involved changes to the collection strategy, sample frame, and content. More information about the redesign is available online.^9^, ^10^, ^11^ The CCHS Public Use Microdata File (PUMF) cycles 2001, 2003, 2005, 2007-2008, 2009-2010, 2011-2012, 2013-2014, 2015-2016, and 2017-2018 were used for this study.

### Ascertainment of risk factors and immigrant status

The current analysis focused on heart disease and 7 cardiovascular risk factors, derived from the CCHS. The risk factors included hypertension, diabetes, obesity, physical inactivity, current smoking, heavy drinking, and high blood cholesterol level.^12^ All measures were based on self-report by the respondents, and they are characterized in Supplemental Table S1.^4^

To ensure consistency across CCHS survey cycles from 2001 to 2018, the *cchsflow* R package (R Foundation, Vienna, Austria) was employed to harmonize the ascertainment of risk factors, renaming variables to a common name.^13^ Derived variables were also created from *cchsflow* that were not available in the original CCHS PUMFs, including binge drinking, physical inactivity, and body mass index (BMI). These variables also are described in the *cchsflow* R package open-license documentation.^14^

Heart disease was defined as any condition that affects the heart and blood vessels. In addition, hypertension, high blood cholesterol level, and diabetes were defined as conditions that were diagnosed by a physician, irrespective of additional treatments. High blood cholesterol level was reported from 2015 onward. BMI was derived from self-reported body mass, in kilograms, divided by the square of height in meters. Obesity was defined as a BMI of ≥ 30.^15^ Smoking status was based on the respondent’s past-year smoking status (current, former, or never). Physical inactivity was defined as leisure-time physical activity < 1.5 metabolic equivalent of task (MET)-hours per day, in which METs are used to describe the intensity of an activity.^16^ Prior to 2015, the activity level of respondents was derived from an aggregate list of self-reported leisure-time physical activities related to the frequency and duration of activity.^17^ Respondents’ average daily energy expenditure during leisure-time physical activity was calculated as the sum of the frequency and duration of the physical activity and the MET value of each activity. From 2015 onward, activity level was separated by age group, as follows: aged 12-17 years, and aged ≥ 18 years. Using the volume-of-weekly-activity variable (PAADVVOL), a derived variable was created by removing the active transportation in the new function to align with previous survey cycles.^18^ Changes in risk factors across survey cycles can be found in Supplemental Table S2.

The respondents were stratified by sex and age groups (aged < 18, 18-49, 50-64, 65-79, or ≥ 80 years), following the age groups of previous analyses.^4^ Six migrant group statuses were generated from ethnicity and immigration status, as follows: nonracialized and non-Indigenous Canadian-born populations; nonracialized and non-Indigenous recent immigrant populations; nonracialized and non-Indigenous established immigrant populations; racialized Canadian-born populations; racialized recent immigrant populations; and racialized established immigrant populations. The recent-immigrants category was comprised of immigrants who have resided in Canada for ≤ 9 years, whereas the established-immigrants category was comprised of those who had resided in Canada for ≥ 10 years. According to Statistics Canada and the Canadian Employment Equity Act, racialized persons were defined as individuals who are underrepresented populations in terms of race, and nonracialized and non-Indigenous persons are defined as individuals who are White.^19^ Reporting for racialized and nonracialized and non-Indigenous groups follows updated recommendations by Statistics Canada.

### Statistical analysis

Risk factor prevalence and trends from 2001 to 2018 were analyzed using linear regression, adjusting for survey sampling weights to represent the Canadian population for each survey cycle. The prevalence estimates were standardized directly for age and sex, using the 2011 Canadian Census population for meaningful comparisons across health behaviours. Time trends in risk factors were stratified by sex, age, and migrant-group statuses. The prevalence of each risk factor was determined across all provinces and territories in Canada. The percent change in risk factor prevalence between the last year of the study period and the baseline in 2001 was calculated to determine relative changes. Using the pooled approach, sampling weights from the combined PUMFs were bootstrapped to assess the variance of prevalence estimates. Statistical uncertainty of prevalence estimates was assessed using survey bootstrap weights for 2015 onward, using the survey R package; bootstrap weights were not available for earlier years. Regression beta-coefficients with *P*-values < 0.05 were considered statistically significant. All analyses were conducted using R 4.0 (R Foundation, Vienna, Austria), and the data and code can be found on GitHub.^20^ The data used in the study are available under the Statistics Canada open license.^21^

## Results

### Study data

A total of 1,065,391 respondents were examined, in the period from 2001 to 2018, a number that was weighted to represent approximately 242 million people in Canada (Supplemental Table S3). The number of respondents was consistent across survey years, as follows: 2001 (n = 59,091); 2003 (n = 58,892); 2005 (n = 56,807); 2007-2008 (n = 56,605); 2009-2010 (n = 54,545); 2011-2012 (n = 53,477); 2013-2014 (n = 54,507); 2015-2016 (n = 45,033); and 2017-2018 (n = 47,101). Across the study period, the largest age group was those aged 18-49 years (51.8%), with 52.6% being male. The largest population subgroup was the nonracialized and non-Indigenous Canadian-born populations (72.0%), followed by racialized established immigrants (8.4%), nonracialized and non-Indigenous established immigrants (7.8%), racialized Canadian-born people (6%), racialized recent immigrants (4.8%), and nonracialized and non-Indigenous recent immigrants (1.3%).

The proportion of racialized Canadian-born individuals increased relative to the total population until 2013, followed by a decrease of 70% from the 2015 survey onward. Similarly, nonracialized, non-Indigenous immigrant groups experienced an initial increase in proportion until 2013, followed by a decrease of 31% from the 2015 survey onward.^22^

### Trends in heart disease

**Figure 1:**
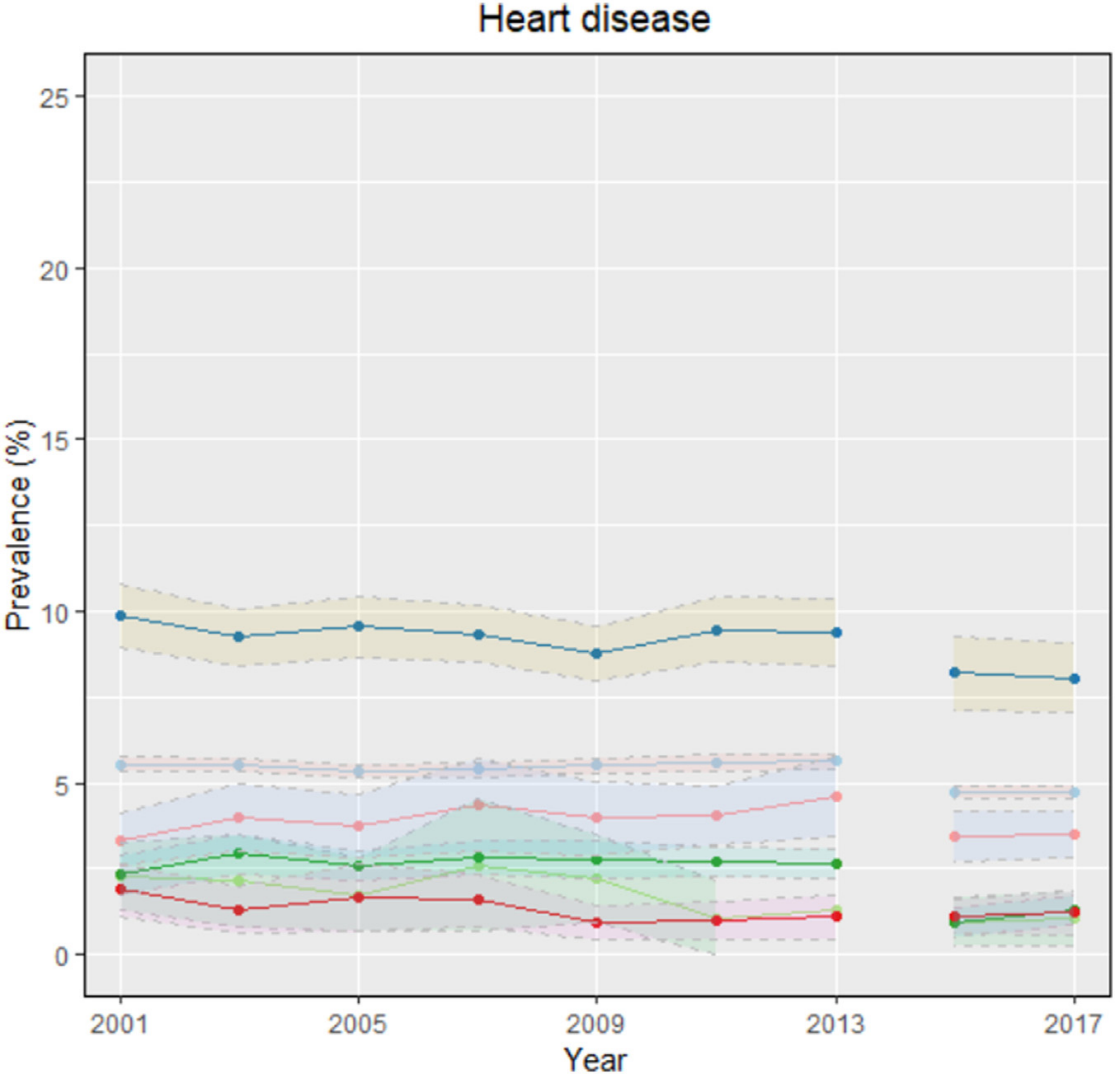
Age- and sex-adjusted prevalence of heart disease from 2001 to 2017/2018, stratified by migrant groups. Nonracialized and non-Indigenous Canadian-born populations—trend line in **light blue,** and 95% confidence interval in **light pink**; nonracialized and non-Indigenous recent immigrant populations—trend line in **light green**, and 95% confidence interval in **light green**; nonracialized and non-Indigenous established immigrant populations—trend line in **dark blue** and 95% confidence interval in **yellow**; racialized Canadian-born populations—trend line in **dark green**, and 95% confidence interval in **light green**; racialized recent immigrant populations—trend line in **red**, and 95% confidence interval in **pink**; and racialized established immigrant populations—trend line in **pink**, and 95% confidence interval in **light blue**. Source: Canadian Community Health Survey (CCHS) —public use micro data. Data harmonized using *cchsflow*. The CCHS underwent a major survey redesign starting in 2015. Bootstrap weights were used for 2015 and 2017. Age and sex standardization were done using the 2011 Canadian Census population.

The prevalence of self-reported heart disease decreased by 21.0% over the 2001-2018 period. A relative decrease in heart disease prevalence of 26.0% and 17.0% was seen in the female and male populations, respectively. The decrease in heart disease prevalence also was apparent in migrant-group statuses, except for racialized established immigrants, a group that had a 4.2% increase (Fig. 1). Compared to 2001, the prevalence of heart disease in 2017 decreased by 44.0% in racialized Canadian-born people, 15.0% in nonracialized and non-Indigenous Canadian-born people, 35.0% in racialized recent immigrants, 53.0% in nonracialized and non-Indigenous recent immigrants, and 19.0% in nonracialized and non-Indigenous established immigrants.

### Trends in risk factors for heart disease

Approximately 70% of the study sample had at least 1 of 7 well-established cardiovascular risk factors (hypertension, diabetes, obesity, physical inactivity, current smoking, heavy drinking, or high blood cholesterol level). The prevalence of the following risk factors increased in Canada over time: diabetes by 54.5%, hypertension by 23.4%, and obesity by 32.3%. From 2001-2018, the prevalence of heavy drinking (–43.8%), current smoking (–38.9%), physical inactivity (–15.4%), and high blood cholesterol level (4.3%) decreased over time (Supplemental Table S4). For both sexes, those aged ≥80 years had the highest prevalence in risk factors in the 2017-2018 cycle, specifically, hypertension (47.4%), diabetes (17.6%), and physical inactivity (77.2%; Supplemental Table S5). In the 2017-2018 cycle, those aged 50-64 years had the highest prevalence of heavy drinking (8.6%), current smoking (18.5%), and obesity (22.6%); those aged 65-79 years had the highest prevalence of high blood cholesterol level (28.7%). By sex, diabetes had a greater difference in prevalence across the period 2001-2018—152.0% in the male population, and 8.6% in the female population, aged < 18 years. The difference in prevalence for physical inactivity was doubled in the female population (–28.1%), compared to the male population (–12.2%), aged < 18 years. In addition, the difference in prevalence for physical inactivity was almost doubled in the male population (43.7%), compared to the female population (22.2%), aged 65-79 years.

### Trends in risk factors for heart disease by provinces and territories

Diabetes doubled in prevalence in all provinces and territories (Supplemental Table S6). Diabetes was most prevalent in New Brunswick, with an 82.7% increase. Although Yukon, Northwestern Territories (NWT), and Nunavut had the lowest prevalence of diabetes and hypertension, they also had the greatest increase in prevalence of hypertension, with 57.5% from 2001 to 2018. Hypertension was most prevalent in New Brunswick, Newfoundland and Labrador, and Nova Scotia, with the greatest increase in New Brunswick, of 45.2%. Physical inactivity decreased across all provinces and territories from 2001 to 2018, with the greatest change seen in Yukon and NWT and Nunavut (–31.7%), Nova Scotia (–27.6%), and Newfoundland and Labrador (–20.1%). Current smoking was the most prevalent in Yukon and NWT and Nunavut in 2001, with the greatest decrease in prevalence of 59.4% from 2001 to 2018. Heavy drinking was least prevalent in Prince Edward Island, but had the greatest difference, of 29.6%. The missing prevalence across certain cycles was attributed to the skip patterns for certain provinces and territories; therefore, British Columbia, Ontario, and Prince Edward Island have valid percent change calculations. For high blood cholesterol level, the greatest difference in prevalence from 2015 to 2018 was seen in Prince Edward Island (–17.3%). Only Alberta (1.9%) and British Columbia (1.7%) had an increase in prevalence across the 2015-2018 time period.

### Trends in risk factors by migrant-group status

**Figure 2:**
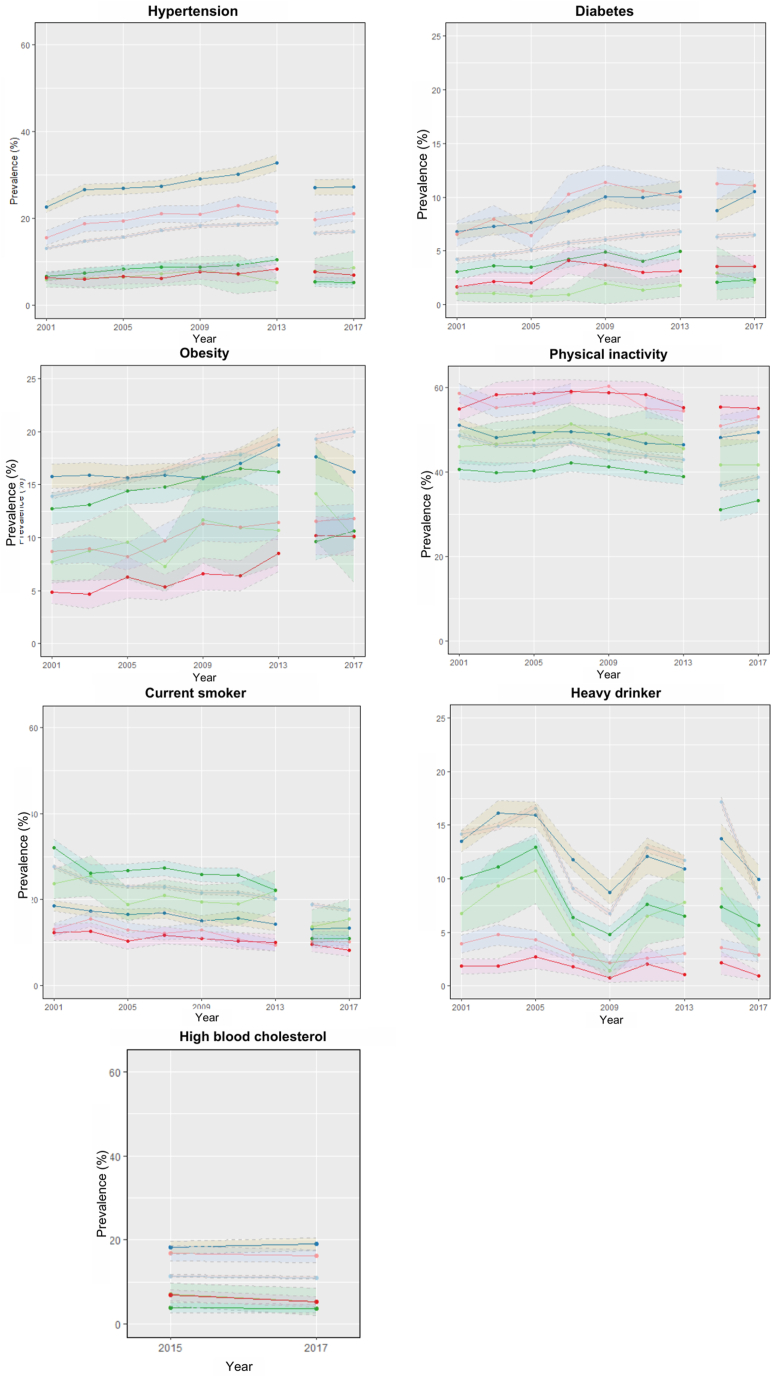
Age- and sex-adjusted risk factor trends from 2001 to 2017/2018, stratified by migrant groups. Nonracialized and non-Indigenous Canadian-born populations—trend line in **light blue**, and 95% confidence interval in **light pink**; nonracialized and non-Indigenous recent immigrant populations—trend line in **light green**, and 95% confidence interval in **light green**; nonracialized and non-Indigenous established immigrant populations—trend line in **dark blue**, and 95% confidence interval in **yellow**; racialized Canadian-born populations—trend line in **dark green,** and 95% confidence interval in **light green**; racialized recent immigrant populations—trend line in **red**, and 95% confidence interval in **pink**; and racialized established immigrant populations—trend line in **pink**, and 95% confidence interval in **light blue**. Source: Canadian Community Health Survey (CCHS)—public use micro data. Data harmonized using *cchsflow*. The CCHS underwent a major survey redesign starting in 2015. Bootstrap weights were used for 2015 and 2017. Age and sex standardization were done using the 2011 Canadian Census population.

Immigrant populations had a higher prevalence of cardiovascular risk factors than that of Canadian-born populations; however, their time trends were similar to nonracialized and non-Indigenous Canadian-born populations (Fig. 2). The trends by migrant-group status were similar for hypertension, diabetes, high blood cholesterol level, and physical inactivity. For diabetes trends, the greatest relative increases were seen in racialized recent immigrants (122.0%) and nonracialized recent immigrants (115.0%). The change in prevalence of heavy drinking was greatest in racialized recent immigrants (–49.7%) and racialized Canadian-born people (–22.2%). Only nonracialized established immigrants had an increased prevalence in heavy drinking (4.3%) from 2001 to 2018. Current smoking decreased across all groups, with the largest decrease in 2015; the greatest decreases were seen in racialized Canadian-born people (–66.1%), and nonracialized and non-Indigenous Canadian-born populations (–36.9%). The prevalence of physical inactivity was higher in immigrants than it was in Canadian-born populations, especially among racialized recent immigrants, with a prevalence > 50% and a relative increase of 0.2%. The greatest change in physical inactivity prevalence was seen in nonracialized and non-Indigenous Canadian-born populations (–19.9%), and racialized Canadian-born populations (–18.0%). The increase in obesity prevalence across 2001-2018 was greatest in racialized recent immigrants (108.0%), nonracialized and non-Indigenous Canadian-born populations (44.0%), and racialized established immigrant populations (36.5%). The greatest change in prevalence of high blood cholesterol level was seen in recent immigrants, both nonracialized and non-Indigenous immigrants (–24.6%), and racialized immigrants (–22.2%).

## Discussion

This national cross-sectional study of the period 2001-2018 described the variations in prevalence of 7 cardiovascular risk factors among over 1 million Canadians, including key subgroups, such as those categorized by ethnicity and immigration status. Between 2001 and 2018, a mixed pattern occurred, with the prevalence of some risk factors decreasing, and that of other risk factors increasing. The self-reported prevalence of heart disease was highest among nonracialized and non-Indigenous established immigrants and among racialized established immigrants in Canada. The prevalences of diabetes, obesity, and hypertension steadily increased over the study period. The overall decrease in heavy alcohol consumption, heavy smoking, physical inactivity, and high blood cholesterol level comes with variations in age groups. Physical inactivity was the most prevalent of all the cardiovascular risk factors.

Heart disease prevalence decreased over the study period, an observation seen in other developed countries that was attributed largely to reduced smoking and improved treatment of hypertension and dyslipidemia.^23^^,^^24^ Treatment for dyslipidemia was recorded for only the recent CCHS cycles, use of statins has been increasing since their introduction in the late 1980s.^25^^,^^26^

Hypertension, diabetes, and obesity are related cardiometabolic risk factors, and their prevalences showed an increasing trend, consistent with that in previous decades, that may result in future increases in CVD.^3^, ^4^, ^5^ Although hypertension has increased, so has treatment with relatively high levels of blood pressure control, compared to that in many countries.^27^ The prevalences of diabetes and obesity increased approximately 1.5-fold over the study period.^4^ Physical inactivity, a related cardiometabolic risk, has decreased in recent years. The findings aligned with those of the Canadian Health Measures Survey (CHMS) from 2007-2017, which collects physical measures for hypertension, obesity, diabetes, and physical inactivity but has a smaller sample size.^25^^,^^28^ Simulation models, such as the Statistics Canada **Po**pulation **He**alth **M**odel (POHEM), can assess the effects of changing risk factors on future CVD incidence.^23^^,^^24^

In this study, immigrants had a higher prevalence of inactivity than their Canadian-born counterparts, which corroborates findings of previous studies.^29^ This disparity can be attributed to socioeconomic status, employment, and social norms relating to physical inactivity. Additionally, the cost associated with certain activities can be an additional deterrent to adopting an active lifestyle.^30^, ^31^, ^32^ Specifically, racialized immigrants exhibited a higher prevalence of physical inactivity.^33^ Established immigrants reported a higher prevalence of risk factors than that of their Canadian-born counterparts. Poorer health outcomes among established immigrants may stem from several factors, such as language concordance and cultural sensitivity of healthcare providers, which can impact the quality and accessibility of healthcare provided to immigrant populations.^34^ Adjei et al. (2020) found that racialized established immigrants had higher odds of developing diabetes, compared to the odds for nonracialized and non-Indigenous Canadian-born citizens, consistent with the results of the present study.^34^ This finding highlights the importance of considering ethnic differences in migrant populations when examining cardiovascular risk factors. With Ontario-landed immigrant data, established immigrants had a significantly higher risk of diabetes, compared to that of recent immigrants.^35^ Consistent with findings of previous studies in Westernized cultures, established immigrants who settled in the US for ≥15 years were more likely to report smoking, obesity, and a high blood cholesterol level than were immigrants who had resided in the US for < 10 years.^36^^,^^37^ The prevalences of obesity, hypertension, and smoking increased with time since immigration to the US, providing support for the healthy-immigrant effect.^37^^,^^38^ This effect describes the fact that recent immigrants report better health than do nonimmigrant populations. However, over time, the health status of recent immigrants declines to the same level as that of their Canadian-born counterparts.^39^^,^^40^ Therefore, the uneven distribution of national cardiovascular risk factors trends among different migrant-group statuses may have significant health implications for cardiovascular outcomes.

### Limitations

Despite the large representative sample, the study poses some limitations. Annual changes in the CCHS questionnaire, response rates, and a major survey redesign in 2015 all affect the findings and their interpretation. However, the observed trends were aligned with findings of other surveys, including the CHMS. Caution is needed when interpreting trends from the 2015 survey period. Further comparison of the population size and characteristics between the CCHS and the Canadian Census is necessary to address potential bias. CCHS relies on self-reported data, resulting in the potential underestimation of risk factors and recall bias. Respondents tend to overreport their healthy behaviours and underreport their unhealthy behaviours in surveys, owing to social-desirability bias. Nonetheless, the self-reported measures in the CCHS were in general alignment with those in the CHMS, and the large sample size of the CCHS allows for subgroup analysis.

Information on immigrants, such as country of origin and detailed ethnicity, is limited in the PUMF, owing to the disclosure control designed to minimize identifying individual respondents, especially for those with lower-frequency demographic characteristics (eg, ethnicity). Using the CCHS PUMF files can showcase the analytical value of these data, and. previous studies using longitudinal data provided evidence to validate the study’s findings.^41^ Future research can link immigration data for a more robust assessment of cardiovascular risk factors in immigrants, considering factors such as country of origin, settlement time, and ethnicity. Further investigation into specific immigrant subgroups, using the CCHS microdata from the Statistics Canada regional data centres and cohort analysis, can help identify at-risk immigrant cohorts.

### Conclusion

This study highlights the cardiovascular risk factors, trends, and disparities among different population groups for the period 2001-2018. Reduced prevalences of smoking and physical inactivity were observed, whereas the increasing prevalences of obesity, diabetes, and hypertension are still evident. Variations in cardiovascular risk factors were found across migrant-group status, particularly among established immigrants in Canada. These findings suggest that migration gaps should be considered in future cardiovascular health strategies in Canada. Effective management of cardiovascular risk factors and equitable access to healthcare services can help reduce the burden of CVD and its risk factors and address disparities among migrant groups.
