# Supplementary material for: Trends in Cardiovascular Risk Factors in Canada: Variation by Migration and Temporal Factors, 2001-2018
Source: CJC Open. 2024 Apr 25;6(8):951–8. doi: 10.1016/j.cjco.2024.04.006 (PMC11357781; doi:10.1016/j.cjco.2024.04.006)
Supplement: Supplementary Material [file mmc1.pdf]

SUPPLEMENTARY MATERIAL

Supplemental Table S1: Risk factor definitions with categories are marked with (*).

Risk factor	Category	Definition
**Hypertension**	Hypertension*	Diagnosis for hypertension
	No hypertension	No hypertension
**Diabetes**	Diabetes*	Diagnosis for diabetes
	No diabetes	No diabetes
**Body Mass Index (BMI)**	Obese*	BMI ≥ 30
	Overweight	BMI from 25 to <30
	Normal weight	BMI from 18.5 to <25
	Underweight	BMI <18.5
**Physical activity**	Inactive*	0 to <1.5 metabolic equivalent of task (MET)-hours/day
	Moderately active	1.5 to <3 MET-hours/day
	Active	≥ 3 MET-hours/day
**Smoking status**	Current*	Daily or occasional consumption of at least 1 cigarette
	Former	Former smokers
	Non-smoker	Never smoked in lifetime
**Alcohol**	Heavy drinker*	≥ 10 (men) or ≥ 6 (women) drinks/week
	Moderate drinker	5 to 9 (men) or 3 to 5 (women) drinks/week
	Light drinker	0 to 4 (men) or 0 to 2 (women) drinks/week
	Non-drinker	No alcohol consumption in the last 12 months
**Blood cholesterol**	High blood cholesterol*	Diagnosis for high blood cholesterol
	No high blood cholesterol	No high blood cholesterol

Supplemental Table S2: Differences in risk factors derived from Canadian Community Health Survey (CCHS) variables pre- and post-2015 survey redesign.

Risk factor	How it was measured before 2015	How it was measured after 2015
**Hypertension**	CCC_071 is a CCHS variable that asked "do you have high blood pressure?"<br><br>Variable name variations were seen across cycles.	Same question was asked but different variable name.
**Diabetes**	CCC_101 is a CCHS variable that asked "do you have diabetes?"<br><br>Variable name variations were seen across cycles.	Same question was asked but different variable name.
**Body Mass Index (BMI)**	We derived BMI from height (HWTGHTM) and weight (HWTGWTK) CCHS variables.<br><br>Variable name variations were seen across cycles.	Same measurements for height and weight were used but different variable name.
**Physical activity[a]**	PACDEE variable is a derived CCHS variable that calculates the daily energy expenditure in leisure time physical activities for all ages.	In CCHS 2015-2018, ages 12-17 and 18+ years old have separate activity variables, where 12-17 year olds use PAY_XXX and 18+ year olds use PAA_XXX. Leisure activity is not directly measured. We used the CCHS derived variable, PAADVVOL, and removed active transportation using a derived function in *cchsflow*. With this function, we combined leisure activity for ages 12+. We calculate the daily energy expenditure which uses the frequency and duration per session of the physical activity as well as the MET value (3 METS for

		leisure and 6 METS for vigorous activity).
**Smoking status**	SMKDSTY is a CCHS derived variable that asked "Type of smoker" with the following categories: daily, occasional, always occasional, former daily, former occasional, and never smoked.<br><br>We grouped the categories into "Current smoker" (daily, occasional, always occasional), "Former "(former daily, former occasional), and "Never smoked".	SMKDVSTY is a CCHS derived variable that asked "Type of smoker" with the following categories: daily, occasional, experimental, former daily, former occasional, and never smoked.<br><br>We grouped the categories into "Current smoker" (daily, occasional), "Former "(experimental, former daily, former occasional), and "Never smoked".
**Alcohol**	We derived alcohol status based on the number of alcoholic drinks per day from Monday to Sunday and sex.	No difference in variables and measurement .
**Blood cholesterol**	Not available in CCHS.	CCC_075 is a CCHS variable that asked "do you have high blood cholesterol or lipids?"

[a] Derivation of physical activity post-2015 survey can be found in *cchsflow*

Supplemental Table S3: Baseline characteristics of unweighted and weighted total CCHS population from 2001 to 2017/2018, stratified by sex. Source: Canadian Community Health Survey (CCHS) - Public Use Micro Data. Data harmonized using *cchsflow*.

Characteristic	Unweighted				Weighted			
	Male, N = 486,058	%	Female N = 579,333	%	Male, N = 119,192,049	%	Female N = 122,686,714	%
**Age**								
<18	55,471	10.8%	53,079	8.7%	12,055,803	9.5%	11,413,200	8.7%
18-49	220,673	42.8%	250,083	40.8%	66,764,054	52.6%	66,503,078	51.0%
50-64	125,071	24.3%	149,015	24.3%	29,305,167	23.1%	29,759,098	22.8%
65-79	89,817	17.4%	116,138	19.0%	15,239,588	12.0%	17,364,375	13.3%
80+	24,062	4.7%	44,353	7.2%	3,578,283	2.8%	5,431,939	4.2%
**Province**								
Alberta	50,812	10.5%	57,397	9.9%	13,129,767	11.0%	12,821,019	10.5%
British Columbia	61,392	12.6%	71,661	12.4%	15,849,440	13.3%	16,261,871	13.3%
Manitoba	27,196	5.6%	32,377	5.6%	4,066,680	3.4%	4,174,808	3.4%
New Brunswick	17,711	3.6%	22,652	3.9%	2,673,386	2.2%	2,818,317	2.3%
Newfoundland and Labrador	14,486	3.0%	17,817	3.1%	1,903,182	1.6%	1,979,509	1.6%
Nova Scotia	18,755	3.9%	23,905	4.1%	3,331,294	2.8%	3,570,157	2.9%
Ontario	154,452	31.8%	185,678	32.1%	45,665,110	38.3%	47,574,287	38.8%
Prince Edward Island	8,206	1.7%	10,696	1.8%	515,659	0.4%	547,409	0.4%
Quebec	94,953	19.5%	112,739	19.5%	28,172,884	23.6%	29,032,400	23.7%
Saskatchewan	27,080	5.6%	32,857	5.7%	3,565,870	3.0%	3,601,526	2.9%
Yukon/Northwest Territories/Nunavut	11,015	2.3%	11,554	2.0%	318,777	0.3%	305,410	0.2%
**Year**								
2001	59,091	12.2%	68,717	11.9%	12,420,321	10.4%	12,803,216	10.4%
2003	58,892	12.1%	69,728	12.0%	12,434,256	10.4%	12,869,919	10.5%

2005	56,807	11.7%	67,633	11.7%	12,269,743	10.3%	12,716,256	10.4%
2007-2008	56,605	11.6%	68,110	11.8%	13,149,308	11.0%	13,563,249	11.1%
2009-2010	54,545	11.2%	65,522	11.3%	13,622,760	11.4%	14,023,559	11.4%
2011-2012	53,477	11.0%	65,605	11.3%	13,767,699	11.6%	14,114,034	11.5%
2013-2014	54,507	11.2%	67,178	11.6%	14,109,744	11.8%	14,449,952	11.8%
2015-2016	45,033	9.3%	52,133	9.0%	13,428,218	11.3%	13,773,453	11.2%
2017-2018	47,101	9.7%	54,707	9.4%	13,990,000	11.7%	14,373,076	11.7%
**Immigration**								
Racialized Canadian-born	29,021	6.0%	32,897	5.7%	7,336,849	6.2%	7,240,579	5.9%
Racialized recent immigrant	10,543	2.2%	12,159	2.1%	5,691,921	4.8%	6,027,981	4.9%
Racialized established immigrant	18,566	3.8%	21,720	3.7%	9,942,146	8.3%	10,382,776	8.5%
Non-racialized Canadian-born	389,886	80.2%	466,337	80.5%	85,344,572	71.6%	87,854,781	71.6%
Non-racialized recent immigrant	3,847	0.8%	4,195	0.7%	1,609,266	1.4%	1,536,921	1.3%
Non-racialized established immigrant	34,195	7.0%	42,025	7.3%	9,267,295	7.8%	9,643,675	7.9%
**Heart disease**								
Heart disease	36,445	7.5%	34,660	6.0%	6,528,336	5.5%	5,071,186	4.1%
No heart disease	448,480	92.3%	543,324	93.8%	112,469,269	94.4%	117,408,315	95.7%
**Body Mass Index (BMI)**								
Underweight	13,347	2.7%	26,788	4.6%	3,275,463	2.7%	6,495,930	5.3%
Normal weight	184,947	38.1%	265,937	45.9%	47,592,640	39.9%	60,498,003	49.3%
Overweight	180,094	37.1%	150,859	26.0%	44,103,272	37.0%	29,603,543	24.1%
Obese	90,485	18.6%	98,021	16.9%	20,243,666	17.0%	17,899,123	14.6%
**Hypertension**								
Hypertension	93,426	19.2%	125,721	21.7%	18,958,788	15.9%	20,209,011	16.5%
No hypertension	390,569	80.4%	452,311	78.1%	99,791,294	83.7%	102,237,932	83.3%
**Blood cholesterol**								

High blood cholesterol	15,114	3.1%	14,210	2.5%	3,774,192	3.2%	3,035,769	2.5%
No high blood cholesterol	67,851	14.0%	84,251	14.5%	21,222,473	17.8%	22,928,366	18.7%
**Diabetes**								
Diabetes	38,764	8.0%	38,945	6.7%	7,742,447	6.5%	6,450,650	5.3%
No diabetes	446,843	91.9%	539,871	93.2%	111,347,340	93.4%	116,136,318	94.7%
**Alcohol**								
Non-drinker	94,381	19.4%	144,543	24.9%	20,646,794	17.3%	28,438,001	23.2%
Light drinker	167,180	34.4%	210,996	36.4%	42,465,412	35.6%	44,743,313	36.5%
Moderate drinker	53,384	11.0%	46,186	8.0%	13,707,303	11.5%	10,496,748	8.6%
Heavy drinker	58,110	12.0%	53,559	9.2%	14,154,900	11.9%	11,937,863	9.7%
**Smoking status**								
Current smoker	114,337	23.5%	113,031	19.5%	27,261,283	22.9%	21,881,835	17.8%
Former smoker	214,882	44.2%	220,184	38.0%	48,754,258	40.9%	42,648,308	34.8%
Never smoked	155,417	32.0%	244,796	42.3%	42,869,016	36.0%	57,910,670	47.2%
**Leisure activity**								
Inactive	206,979	42.6%	287,058	49.5%	50,056,613	42.0%	59,808,744	48.7%
Moderately active	96,942	19.9%	123,632	21.3%	23,242,916	19.5%	25,106,187	20.5%
Active	161,013	33.1%	154,399	26.7%	41,004,344	34.4%	34,171,985	27.9%
**Number of risk factors**								
0	136,964	28.2%	156,879	27.1%	35,953,046	30.2%	36,458,266	29.7%
1	168,448	34.7%	210,240	36.3%	42,682,387	35.8%	47,373,734	38.6%
2+	180,646	37.2%	212,214	36.6%	40,556,615	34.0%	38,854,713	31.7%

Supplemental Table S4: Age- and sex-standardized prevalence of risk factors for survey respondents aged 12 years and older, from 2001 to 2017/2018. Source: Canadian Community Health Survey (CCHS) - Public Use Micro Data. Data harmonized using *cchsflow*. The CCHS underwent a major survey redesign starting in 2015. Bootstrap weights were used for 2015 and 2017. Age and sex standardization were done using the 2011 Canadian Census population.

	Year, prevalence (%, 95% CI)									Relative change, %	p-value
Risk factor	2001	2003	2005	2007	2009	2011	2013	2015	2017		
**Both sexes**											
Hypertension	13.6 (13.3,13.8)	15.4 (15.1,15.7)	16.1 (15.8,16.4)	17.3 (16.9,17.6)	18.2 (17.8,18.5)	18.5 (18.2, 18.9)	18.9 (18.5,19.3)	16.4 (16.1,16.8)	16.7 (16.4,17.1)	23.4	0.08
Diabetes	4.4 (4.2,4.6)	4.9 (4.7,5.1)	5.1 (5.0,5.3)	6.1 (5.9,6.4)	6.6 (6.4,6.8)	6.7 (6.5, 7.0)	7.0 (6.7,7.2)	6.6 (6.3,6.8)	6.8 (6.6,7.0)	54.5	< 0.01
Obesity	13.3 (13,13.6)	13.9 (13.6,14.2)	14.7 (14.4,15.0)	15.0 (14.6,15.3)	16 (15.7,16.4)	16.4 (16.0, 16.8)	17.5 (17.1,17.9)	17.3 (16.9,17.7)	17.6 (17.2,18.0)	32.3	< 0.01
Physical inactivity	49.4 (49,49.8)	47.3 (46.8,47.7)	47.2 (46.7,47.6)	48.4 (47.9,48.8)	47 (46.5,47.5)	45.6 (45.0, 46.1)	44.8 (44.2,45.3)	39.9 (39.3,40.4)	41.8 (41.2,42.3)	-15.4	< 0.01
Current smoker	25.4 (25.1,25.8)	22.7 (22.3,23.0)	21.8 (21.4,22.1)	21.3 (20.9,21.7)	20.1 (19.7,20.5)	19.8 (19.4, 20.2)	18.3 (17.9,18.7)	16.6 (16.2,17.0)	15.5 (15.2,15.9)	-38.9	< 0.01
Heavy drinker	12.7 (12.5,13)	13.6 (13.3,13.9)	15.1 (14.8,15.4)	8.3 (8.0,8.5)	6.1 (5.8,6.3)	10.9 (10.5, 11.2)	9.8 (9.5,10.1)	14.1 (13.7,14.4)	7.2 (6.9,7.4)	-43.8	0.28
High blood cholesterol	-	-	-	-	-	-	-	11.8 (11.5,12.1)	11.3 (11.0,11.6)	-4.3	NaN
**Male**											
Hypertension	12.1 (11.7,12.5)	14.1 (13.7,14.6)	15.1 (14.7,15.5)	16.5 (16.0,16.9)	17.7 (17.2,18.3)	18.2 (17.6, 18.8)	19.4 (18.8,20.0)	16.5 (16.0,17.0)	17.2 (16.7,17.7)	41.9	0.01
Diabetes	4.7 (4.5,5.0)	5.3 (5.0,5.5)	5.6 (5.4,5.9)	6.7 (6.3,7.1)	7.6 (7.2,7.9)	7.2 (6.9, 7.6)	7.7 (7.3,8.1)	7.3 (6.9,7.6)	7.8 (7.5,8.2)	65.5	< 0.01
Obesity	13.8 (13.4,14.2)	14.6 (14.2,15.1)	16.0 (15.6,16.5)	16.2 (15.6,16.7)	17.6 (17.0,18.2)	17.4 (16.8, 18.0)	19.1 (18.5,19.7)	18.9 (18.3,19.5)	18.8 (18.2,19.4)	36.3	< 0.01
Physical inactivity	44.2 (43.6,44.8)	43.7 (43.1,44.4)	44.3 (43.7,45.0)	44.6 (43.9,45.3)	43.3 (42.6,44.1)	42.9 (42.1, 43.8)	41.9 (41.1,42.7)	35.8 (35.0,36.5)	38.0 (37.2,38.7)	-14.2	0.01

Current smoker	27.6 (27.1,28.1)	24.7 (24.2,25.3)	23.5 (23.0,24.1)	24.1 (23.5,24.7)	23.0 (22.4,23.7)	22.3 (21.6, 23.0)	21.3 (20.6,21.9)	19.4 (18.8,20.0)	18.5 (17.9,19.1)	-32.8	< 0.01
Heavy drinker	14.3 (13.9,14.7)	15.4 (15.0,15.9)	17.3 (16.8,17.7)	9.2 (8.8,9.6)	6.9 (6.5,7.3)	11.6 (11.1, 12.1)	10.4 (9.9,10.8)	15.3 (14.7,15.8)	7.4 (7.0,7.9)	-48.0	0.19
High blood cholesterol	-	-	-	-	-	-	-	13.2 (12.7,13.6)	12.8 (12.3,13.2)	-3.0	NaN
**Female**											
Hypertension	14.9 (14.5,15.3)	16.5 (16.1,17.0)	17.0 (16.6,17.4)	18.0 (17.6,18.5)	18.6 (18.1,19.1)	18.9 (18.4, 19.4)	18.3 (17.8,18.9)	16.4 (15.9,16.8)	16.3 (15.8,16.8)	9.3	0.44
Diabetes	4.1 (3.9,4.3)	4.5 (4.3,4.8)	4.7 (4.4,4.9)	5.6 (5.3,5.9)	5.7 (5.4,6.0)	6.2 (5.8, 6.6)	6.3 (6.0,6.6)	5.9 (5.6,6.3)	5.8 (5.5,6.1)	42.5	< 0.01
Obesity	12.8 (12.5,13.2)	13.3 (12.9,13.7)	13.4 (13.0,13.8)	13.8 (13.4,14.2)	14.6 (14.1,15.0)	15.4 (14.9, 15.9)	16.0 (15.5,16.5)	15.7 (15.2,16.2)	16.5 (16.0,17.0)	28.3	< 0.01
Physical inactivity	54.3 (53.7,54.8)	50.6 (50.0,51.2)	49.8 (49.2,50.4)	51.9 (51.3,52.6)	50.5 (49.8,51.2)	48.1 (47.4, 48.8)	47.5 (46.8,48.2)	43.8 (43.0,44.5)	45.4 (44.7,46.1)	-16.4	< 0.01
Current smoker	23.4 (22.9,23.8)	20.7 (20.2,21.2)	20.1 (19.6,20.5)	18.7 (18.2,19.2)	17.3 (16.8,17.8)	17.4 (16.8, 17.9)	15.5 (15.0,16.0)	14.0 (13.5,14.5)	12.7 (12.2,13.1)	-45.8	< 0.01
Heavy drinker	11.2 (10.9,11.6)	11.9 (11.5,12.3)	13.1 (12.8,13.5)	7.3 (7.0,7.7)	5.3 (5.0,5.6)	10.2 (9.7, 10.6)	9.2 (8.8,9.6)	12.9 (12.4,13.4)	6.9 (6.5,7.3)	-38.7	0.41
High blood cholesterol	-	-	-	-	-	-	-	10.5 (10.1,10.9)	9.9 (9.5,10.3)	-6.0	NaN

Supplemental Table S5: Sex-standardized prevalence of cardiovascular risk factors by age from 2001 to 2017/2018. Source: Canadian Community Health Survey (CCHS) - Public Use Micro Data. Data harmonized using *cchsflow*. The CCHS underwent a major survey redesign starting in 2015. Bootstrap weights were used for 2015 and 2017. Sex standardization was done using the 2011 Canadian Census population.

Risk factor; sex; age	Year, prevalence (%, 95% CI)									Relative change, %	p-value
	2001	2003	2005	2007	2009	2011	2013	2015	2017		
**Hypertension**											
Both sexes											
<18	0.6 (0.4,0.8)	0.6 (0.4,0.8)	0.4 (0.2,0.5)	0.4 (0.3,0.6)	0.5 (0.3,0.7)	0.4 (0.2,0.6)	0.4 (0.3,0.6)	0.7 (0.4,1)	0.7 (0.3,1.0)	4.8	0.52
18-49	5.3 (5.0,5.5)	5.7 (5.3,6.0)	5.5 (5.2,5.7)	5.7 (5.4,6.0)	5.8 (5.4,6.2)	5.7 (5.3,6.1)	5.6 (5.2,6.0)	5.7 (5.3,6.1)	5.4 (5.0,5.8)	2.1	< 0.01
50-64	22.3 (21.5,23.1)	25.2 (24.4,26.0)	25.8 (25.0,26.6)	27.0 (26.1,27.8)	27.1 (26.1,28.0)	26.7 (25.7,27.7)	26.7 (25.7,27.7)	24.6 (23.7,25.5)	25.8 (24.9,26.8)	15.9	< 0.01
65-79	38.3 (37.2,39.3)	42.2 (41.2,43.2)	43.5 (42.5,44.5)	45.4 (44.3,46.5)	47.3 (46.2,48.4)	47.2 (46.1,48.4)	46.3 (45.3,47.4)	43.1 (42.0,44.2)	42.0 (41.0,43.1)	9.8	< 0.01
80+	39.9 (37.9,41.9)	44.9 (43.0,46.7)	47.7 (45.9,49.5)	51.5 (49.7,53.3)	52.4 (50.4,54.3)	52.5 (50.6,54.3)	52.6 (50.9,54.4)	49.7 (47.7,51.7)	47.4 (45.5,49.3)	18.7	< 0.01
Male											
<18	0.7 (0.4,1.0)	0.6 (0.4,0.8)	0.3 (0.1,0.4)	0.5 (0.3,0.7)	0.6 (0.3,0.9)	0.4 (0.2,0.7)	0.6 (0.3,0.9)	0.6 (0.3,0.9)	0.7 (0.1,1.3)	5.8	0.62
18-49	5.6 (5.2,5.9)	6.3 (5.9,6.8)	6.1 (5.7,6.6)	6.4 (5.9,6.8)	6.7 (6.1,7.3)	6.8 (6.2,7.4)	6.9 (6.2,7.6)	6.8 (6.2,7.4)	6.4 (5.8,6.9)	14.4	< 0.01
50-64	20.7 (19.6,21.8)	24.6 (23.4,25.7)	25.9 (24.8,27.1)	27.0 (25.7,28.3)	28.4 (27.0,29.9)	27.5 (26.0,28.9)	29.4 (27.9,30.9)	26.4 (25.1,27.7)	28.6 (27.2,30.0)	38.1	< 0.01
65-79	34.2 (32.7,35.8)	38.1 (36.5,39.6)	40.1 (38.6,41.6)	42.5 (40.9,44.1)	44.4 (42.8,45.9)	46.0 (44.2,47.8)	46.2 (44.6,47.8)	42.0 (40.4,43.7)	42.2 (40.7,43.7)	23.2	< 0.01
80+	32.8 (29.6,36.1)	33.7 (30.8,36.6)	38.5 (35.6,41.4)	45.0 (42.0,48.1)	44.3 (41.4,47.3)	46.0 (43.1,48.9)	48.9 (46.0,51.7)	43.0 (39.8,46.1)	44.1 (41.0,47.2)	34.4	< 0.01
Female											

<18	0.6 (0.4,0.7)	0.7 (0.4,1.0)	0.5 (0.2,0.7)	0.4 (0.2,0.6)	0.4 (0.2,0.7)	0.4 (0.1,0.7)	0.3 (0.1,0.5)	0.8 (0.3,1.2)	0.6 (0.3,0.9)	3.6	0.52
18-49	5.0 (4.7,5.3)	5.0 (4.6,5.4)	4.8 (4.4,5.2)	5.0 (4.6,5.4)	5.0 (4.4,5.5)	4.6 (4.2,5.1)	4.3 (3.8,4.8)	4.6 (4.1,5.0)	4.4 (3.9,4.9)	-11.0	< 0.01
50-64	23.8 (22.7,24.9)	25.9 (24.9,27.0)	25.7 (24.7,26.8)	26.9 (25.8,28.1)	25.7 (24.5,26.9)	26.0 (24.6,27.4)	24.1 (22.8,25.4)	22.8 (21.5,24.1)	23.2 (21.9,24.5)	-2.7	< 0.01
65-79	41.7 (40.3,43.2)	45.9 (44.5,47.2)	46.5 (45.2,47.9)	48.1 (46.6,49.5)	50.1 (48.6,51.5)	48.4 (47.0,49.9)	46.5 (45.1,47.8)	44.1 (42.6,45.6)	41.8 (40.4,43.3)	0.3	< 0.01
80+	44.4 (41.9,46.9)	51.2 (48.9,53.5)	53.2 (51.0,55.5)	55.9 (53.6,58.1)	57.4 (54.9,59.9)	57.1 (54.7,59.6)	55.3 (53,57.6)	54.7 (52.2,57.2)	50 (47.6,52.3)	12.6	< 0.01
**Diabetes**											
Both sexes											
<18	0.3 (0.2,0.4)	0.4 (0.3,0.5)	0.3 (0.2,0.4)	0.3 (0.2,0.5)	0.5 (0.3,0.7)	0.4 (0.3,0.6)	0.4 (0.2,0.5)	0.5 (0.3,0.7)	0.5 (0.3,0.8)	71.0	0.46
18-49	1.7 (1.6,1.9)	1.7 (1.5,1.9)	1.7 (1.6,1.9)	2.0 (1.8,2.2)	2.0 (1.8,2.3)	2.1 (1.9,2.4)	2.1 (1.9,2.3)	2.4 (2.1,2.7)	2.3 (2.1,2.5)	35.9	0.04
50-64	7.1 (6.7,7.6)	8.3 (7.8,8.8)	8.1 (7.7,8.6)	9.9 (9.2,10.6)	9.6 (9.0,10.2)	9.5 (8.9,10.2)	9.8 (9.2,10.4)	9.7 (9.0,10.4)	10.3 (9.7,11.0)	44.7	< 0.01
65-79	13.1 (12.4,13.8)	14.0 (13.3,14.8)	14.7 (13.9,15.4)	16.2 (15.4,17.0)	17.8 (17.0,18.7)	17.8 (17.0,18.7)	18.0 (17.2,18.9)	17.6 (16.7,18.5)	17.7 (16.9,18.5)	35.0	< 0.01
80+	11.3 (10.1,12.5)	11.4 (10.3,12.5)	13.5 (12.3,14.6)	16.2 (14.7,17.7)	19.1 (17.3,20.9)	16.9 (15.5,18.4)	16.6 (15.2,18.0)	18.1 (16.5,19.7)	17.6 (16.1,19.0)	55.6	< 0.01
Male											
<18	0.3 (0.1,0.4)	0.4 (0.2,0.6)	0.3 (0.2,0.5)	0.4 (0.2,0.6)	0.6 (0.2,1)	0.6 (0.3,0.9)	0.3 (0.1,0.6)	0.5 (0.2,0.9)	0.7 (0.3,1.1)	152.0	0.49
18-49	1.7 (1.5,1.9)	1.6 (1.4,1.8)	1.8 (1.6,2.0)	2.0 (1.7,2.3)	2.2 (1.9,2.5)	2.2 (1.8,2.6)	2.4 (2.0,2.7)	2.7 (2.3,3.2)	2.4 (2.1,2.7)	43.4	0.09
50-64	8.3 (7.5,9.0)	9.7 (8.9,10.6)	9.5 (8.7,10.2)	11.5 (10.3,12.6)	11.4 (10.4,12.4)	10.5 (9.6,11.4)	11.2 (10.2,12.2)	11.2 (10.3,12.1)	12.5 (11.5,13.6)	51.7	< 0.01
65-79	14.9 (13.7,16.1)	16 (14.8,17.1)	17.1 (15.8,18.3)	18.7 (17.4,20.0)	21.7 (20.3,23.1)	20.5 (19.2,21.8)	20.9 (19.6,22.2)	20.0 (18.6,21.3)	21.3 (20.1,22.6)	43.1	< 0.01
80+	14.1 (11.8,16.3)	12.8 (10.9,14.7)	16.0 (13.8,18.1)	17.9 (15.3,20.4)	22.3 (19.7,25.0)	19.9 (17.5,22.2)	17.7 (15.6,19.8)	20.5 (17.8,23.1)	22.2 (19.7,24.8)	58.2	< 0.01

Female											
<18	0.4 (0.2,0.5)	0.3 (0.2,0.4)	0.3 (0.1,0.4)	0.3 (0.1,0.4)	0.4 (0.2,0.6)	0.3 (0.1,0.5)	0.4 (0.2,0.6)	0.5 (0.2,0.8)	0.4 (0.1,0.6)	8.6	0.47
18-49	1.7 (1.5,2)	1.7 (1.5,2)	1.7 (1.5,1.9)	2 (1.8,2.3)	1.9 (1.6,2.1)	2.1 (1.7,2.4)	1.9 (1.6,2.1)	2.1 (1.7,2.4)	2.2 (1.9,2.6)	28.7	0.02
50-64	6 (5.4,6.6)	7 (6.3,7.6)	6.9 (6.3,7.4)	8.3 (7.5,9.1)	7.9 (7.1,8.6)	8.7 (7.7,9.7)	8.4 (7.6,9.2)	8.3 (7.3,9.3)	8.2 (7.4,9)	35.4	< 0.01
65-79	11.5 (10.6,12.4)	12.3 (11.4,13.3)	12.5 (11.7,13.4)	14 (13,15)	14.2 (13.2,15.1)	15.4 (14.3,16.5)	15.4 (14.3,16.4)	15.3 (14.2,16.5)	14.2 (13.2,15.2)	23.6	< 0.01
80+	9.5 (8.1,11)	10.6 (9.3,12)	12 (10.7,13.3)	15.1 (13.2,16.9)	17 (14.6,19.4)	14.8 (13,16.6)	15.8 (14.1,17.6)	16.3 (14.4,18.2)	14 (12.4,15.5)	46.2	< 0.01
**Obesity**											
Both sexes											
<18	3.6 (3.3,4)	3.4 (3.1,3.8)	3.3 (2.9,3.8)	1.8 (1.5,2.2)	2.7 (2.3,3.1)	3 (2.4,3.5)	3.4 (2.9,4)	2.9 (2.4,3.4)	3.3 (2.7,3.8)	-10.2	< 0.01
18-49	13.3 (12.9,13.7)	13.7 (13.2,14.1)	14.1 (13.7,14.5)	14.3 (13.8,14.8)	15.2 (14.7,15.8)	15.7 (15.1,16.2)	16.8 (16.2,17.4)	16.7 (16.1,17.3)	17.2 (16.6,17.8)	29.4	< 0.01
50-64	18.6 (17.8,19.3)	19.7 (19,20.4)	20.3 (19.5,21)	20.5 (19.7,21.3)	21.6 (20.8,22.5)	22 (21,22.9)	22.7 (21.8,23.6)	22.6 (21.7,23.5)	22.6 (21.7,23.5)	21.8	< 0.01
65-79	15.4 (14.6,16.1)	16.3 (15.6,17.1)	16.8 (16,17.5)	17.9 (17.1,18.7)	19 (18.2,19.8)	18.6 (17.8,19.4)	19.9 (19.1,20.6)	20.2 (19.3,21)	20.2 (19.4,21)	31.8	< 0.01
80+	9.1 (7.9,10.3)	8.7 (7.8,9.7)	9.9 (8.8,11)	9.3 (8.3,10.3)	9.9 (8.9,11)	9.3 (8.4,10.2)	11.1 (9.9,12.3)	9.3 (8.3,10.4)	10.4 (9.3,11.4)	13.9	< 0.01
Male											
<18	4.4 (3.8,5.1)	4.2 (3.6,4.8)	3.9 (3.2,4.6)	2.6 (2,3.2)	3.3 (2.6,3.9)	3.9 (2.9,4.8)	4.3 (3.4,5.2)	3.8 (3,4.7)	3.4 (2.6,4.2)	-22.8	< 0.01
18-49	14.2 (13.7,14.8)	14.9 (14.3,15.6)	16.2 (15.5,16.8)	15.8 (15.1,16.5)	17.5 (16.7,18.4)	17.3 (16.4,18.2)	19.2 (18.2,20.2)	18.3 (17.4,19.2)	18.3 (17.4,19.2)	28.4	< 0.01
50-64	18.9 (17.9,19.9)	20.1 (19,21.2)	21.4 (20.3,22.4)	22 (20.8,23.3)	23.3 (22,24.6)	22.9 (21.5,24.3)	24.2 (22.8,25.6)	25.2 (23.9,26.6)	25.1 (23.7,26.4)	32.6	< 0.01
65-79	14.5 (13.3,15.6)	15.6 (14.5,16.7)	16.7 (15.6,17.8)	18.4 (17.2,19.7)	18.8 (17.6,20.1)	18.4 (17.2,19.7)	20.3 (19.1,21.4)	20.8 (19.5,22.1)	20.8 (19.6,21.9)	43.7	< 0.01

80+	7.5 (5.9,9.1)	7.6 (6,9.2)	8.7 (7,10.5)	7.2 (5.9,8.6)	8.8 (7.2,10.3)	8.3 (6.9,9.8)	9.6 (8.1,11.1)	9.1 (7.6,10.5)	9.1 (7.6,10.5)	21.1	< 0.01
Female											
<18	2.8 (2.4,3.3)	2.6 (2.2,3.1)	2.7 (2.1,3.3)	1.1 (0.7,1.5)	2.2 (1.7,2.7)	2.0 (1.4,2.6)	2.5 (1.9,3.1)	2.0 (1.5,2.5)	3.1 (2.4,3.9)	9.9	< 0.01
18-49	12.4 (11.9,12.9)	12.5 (11.8,13.1)	12.1 (11.6,12.6)	12.8 (12.2,13.4)	13.1 (12.4,13.8)	14.1 (13.3,14.8)	14.4 (13.7,15.2)	15.1 (14.4,15.9)	16.2 (15.4,17)	30.7	< 0.01
50-64	18.2 (17.2,19.2)	19.3 (18.4,20.3)	19.2 (18.3,20.2)	19.0 (18.1,20.0)	20.0 (18.9,21.1)	21.1 (19.8,22.3)	21.2 (20.1,22.4)	20 (18.9,21.2)	20.2 (19.1,21.3)	10.8	< 0.01
65-79	16.2 (15.1,17.2)	17.0 (16.0,18.0)	16.9 (15.9,17.8)	17.5 (16.4,18.5)	19.1 (18.0,20.2)	18.8 (17.7,19.8)	19.5 (18.5,20.5)	19.6 (18.5,20.7)	19.7 (18.7,20.8)	22.2	< 0.01
80+	10.1 (8.5,11.8)	9.4 (8.1,10.6)	10.6 (9.3,12.0)	10.7 (9.3,12.0)	10.6 (9.3,12.0)	10.0 (8.8,11.2)	12.1 (10.4,13.8)	9.5 (8.1,10.9)	11.4 (9.9,12.9)	12.5	< 0.01
**Physical inactivity**											
Both sexes											
<18	27.7 (26.7,28.7)	27.3 (26.3,28.3)	26.1 (25.0,27.3)	26.8 (25.4,28.2)	26.8 (25.5,28.2)	25.9 (24.5,27.4)	27.0 (25.5,28.6)	20.9 (19.4,22.4)	21.6 (20.1,23.0)	-22.1	< 0.01
18-49	50.8 (50.2,51.3)	47.8 (47.1,48.5)	46.9 (46.3,47.5)	48.4 (47.7,49.1)	45.9 (45.2,46.7)	44.8 (44.0,45.6)	44.2 (43.3,45.1)	34.6 (33.8,35.4)	36.3 (35.4,37.1)	-28.6	< 0.01
50-64	53.4 (52.4,54.3)	51.7 (50.7,52.6)	51.3 (50.4,52.3)	52.0 (51.1,53.0)	51.0 (49.9,52.1)	48.6 (47.4,49.8)	48.0 (46.9,49.2)	42.4 (41.4,43.5)	44.6 (43.5,45.7)	-16.5	< 0.01
65-79	53.4 (52.3,54.5)	51.7 (50.7,52.8)	50.4 (49.4,51.4)	52.1 (51.0,53.2)	51.6 (50.5,52.6)	49.1 (48.0,50.3)	46.6 (45.5,47.6)	55.4 (54.3,56.4)	56.5 (55.5,57.6)	5.8	< 0.01
80+	65.1 (63.0,67.1)	62.9 (61.1,64.8)	63.5 (61.7,65.3)	60.6 (58.8,62.4)	60.9 (58.9,62.8)	60.1 (58.3,61.9)	54.8 (53.0,56.6)	76.1 (74.5,77.8)	77.2 (75.5,78.8)	18.6	< 0.01
Male											
<18	20.8 (19.5,22.0)	21.9 (20.6,23.2)	20.5 (19.0,22.0)	20.8 (19.1,22.6)	21.2 (19.4,23.0)	21.6 (19.7,23.4)	22.2 (20.2,24.2)	17.2 (15.4,19.1)	18.2 (16.4,20.0)	-12.2	< 0.01
18-49	47.0 (46.2,47.9)	45.4 (44.4,46.3)	45.5 (44.6,46.4)	45.4 (44.4,46.4)	42.5 (41.4,43.6)	43.3 (42.1,44.5)	42.0 (40.7,43.2)	30.2 (29.1,31.4)	32.5 (31.4,33.7)	-30.8	< 0.01
50-64	50.2 (48.8,51.5)	50.8 (49.4,52.2)	50.8 (49.4,52.2)	50.5 (49.0,52.0)	50.2 (48.6,51.8)	47.1 (45.4,48.9)	47.3 (45.6,49.0)	41.0 (39.4,42.5)	42.4 (40.8,44.0)	-15.4	< 0.01

65-79	44.4 (42.8,46.0)	44.5 (42.9,46.1)	44.0 (42.5,45.5)	45.8 (44.2,47.4)	45.8 (44.2,47.4)	44.6 (42.8,46.4)	41.0 (39.4,42.5)	51.8 (50.1,53.4)	53.0 (51.4,54.5)	19.3	< 0.01
80+	53.5 (49.9,57.1)	52.0 (48.8,55.1)	52.8 (49.8,55.7)	50.8 (47.8,53.9)	52.3 (49.3,55.3)	50.9 (48.0,53.9)	48.0 (45.2,50.9)	72.1 (69.3,74.9)	74.4 (71.7,77.0)	39.0	< 0.01
Female											
<18	34.7 (33.3,36.2)	32.7 (31.1,34.2)	31.9 (30.2,33.7)	32.7 (30.6,34.8)	32.4 (30.4,34.3)	30.4 (28.2,32.6)	32.1 (29.8,34.4)	24.6 (22.3,27.0)	25.0 (22.7,27.2)	-28.1	< 0.01
18-49	54.3 (53.5,55.1)	50.2 (49.2,51.1)	48.3 (47.4,49.1)	51.2 (50.3,52.2)	49.2 (48.2,50.3)	46.2 (45.1,47.4)	46.4 (45.2,47.5)	38.9 (37.7,40.0)	39.8 (38.7,41.0)	-26.7	< 0.01
50-64	56.4 (55.2,57.7)	52.5 (51.3,53.7)	51.8 (50.6,53.1)	53.6 (52.3,54.8)	51.8 (50.3,53.2)	50.0 (48.4,51.5)	48.7 (47.1,50.3)	43.9 (42.4,45.4)	46.6 (45.1,48.2)	-17.3	< 0.01
65-79	61.3 (59.8,62.7)	58.1 (56.8,59.5)	56.1 (54.8,57.5)	57.8 (56.4,59.3)	57.0 (55.6,58.4)	53.2 (51.8,54.7)	51.8 (50.5,53.2)	58.7 (57.3,60.2)	59.9 (58.5,61.3)	-2.2	< 0.01
80+	72.3 (69.9,74.7)	69.1 (66.9,71.3)	70.1 (67.8,72.3)	67.2 (65.0,69.4)	66.2 (63.6,68.8)	66.7 (64.4,69.0)	59.6 (57.2,61.9)	79.1 (77.2,81.1)	79.4 (77.3,81.4)	9.7	< 0.01
**Current smoker**											
Both sexes											
<18	18.7 (17.9,19.5)	14.7 (13.9,15.5)	8.1 (7.4,8.7)	7.9 (7.2,8.6)	7.3 (6.5,8.0)	5.8 (5.0,6.5)	4.4 (3.8,5.0)	3.5 (2.8,4.2)	3.2 (2.5,3.8)	-83.1	< 0.01
18-49	32.0 (31.5,32.5)	28.9 (28.3,29.5)	28.0 (27.5,28.6)	27.0 (26.4,27.6)	25.0 (24.3,25.6)	25.0 (24.2,25.7)	23.1 (22.4,23.8)	19.4 (18.8,20.1)	18.1 (17.5,18.7)	-43.4	< 0.01
50-64	23.4 (22.6,24.1)	21.5 (20.8,22.3)	21.1 (20.3,21.8)	21.7 (20.9,22.5)	21.6 (20.8,22.5)	21.7 (20.7,22.6)	20.0 (19.1,20.8)	19.8 (19.0,20.7)	18.5 (17.7,19.3)	-20.9	< 0.01
65-79	13.7 (13.0,14.4)	12.1 (11.4,12.7)	12.1 (11.5,12.7)	12.2 (11.5,13.0)	11.7 (11.0,12.3)	11.0 (10.3,11.7)	11.1 (10.5,11.7)	11.0 (10.3,11.7)	10.8 (10.2,11.5)	-20.8	0.02
80+	6.3 (5.2,7.3)	6.3 (5.3,7.4)	5.9 (5.1,6.8)	5.2 (4.4,5.9)	5.1 (4.3,5.9)	5.0 (4.1,5.8)	4.7 (4.0,5.4)	3.6 (3.0,4.2)	4.7 (3.7,5.6)	-26.1	0.06
Male											
<18	17.7 (16.5,18.9)	14.3 (13.2,15.4)	7.1 (6.2,7.9)	8.1 (7.1,9.2)	7.4 (6.4,8.4)	5.6 (4.5,6.6)	4.8 (3.8,5.7)	3.8 (2.7,4.8)	2.9 (2.2,3.7)	-83.4	< 0.01
18-49	35.0 (34.2,35.8)	31.9 (31.0,32.9)	30.8 (30.0,31.6)	30.9 (29.9,31.8)	28.8 (27.8,29.8)	28.7 (27.6,29.8)	27.6 (26.5,28.7)	23.0 (22.0,24.0)	22.3 (21.4,23.3)	-36.2	< 0.01

50-64	24.9 (23.7,26.0)	22.9 (21.7,24)	21.9 (20.8,23)	23.5 (22.3,24.7)	24.5 (23.1,25.9)	23.7 (22.2,25.1)	22.0 (20.6,23.3)	22.3 (21.0,23.5)	20.8 (19.6,22.1)	-16.1	< 0.01
65-79	14.5 (13.4,15.6)	12.3 (11.3,13.2)	12.3 (11.4,13.3)	13.5 (12.3,14.7)	12.5 (11.5,13.6)	11.2 (10.0,12.4)	11.9 (10.9,12.9)	12.3 (11.2,13.4)	11.9 (10.9,12.9)	-18.0	< 0.01
80+	7.5 (5.4,9.6)	7.5 (5.8,9.2)	6.6 (4.8,8.3)	5.6 (4.3,6.8)	5.6 (4.1,7.2)	5.7 (4.2,7.1)	5.4 (4.2,6.6)	3.0 (2.2,3.8)	5.1 (3.3,6.9)	-32.1	0.13
Female											
<18	19.7 (18.5,20.9)	15.2 (14.1,16.3)	9.1 (8.1,10.1)	7.6 (6.6,8.7)	7.2 (6.1,8.2)	6.0 (4.8,7.1)	4.0 (3.3,4.8)	3.3 (2.4,4.1)	3.4 (2.3,4.5)	-82.8	< 0.01
18-49	29.1 (28.4,29.8)	26.0 (25.2,26.8)	25.3 (24.6,26.1)	23.3 (22.6,24.1)	21.3 (20.5,22.1)	21.3 (20.4,22.2)	18.8 (17.9,19.6)	16.0 (15.2,16.7)	14.0 (13.3,14.7)	-51.8	< 0.01
50-64	21.9 (20.9,23.0)	20.2 (19.3,21.2)	20.3 (19.3,21.2)	20.0 (19.0,21.0)	18.8 (17.8,19.9)	19.8 (18.6,21.0)	18.1 (17.1,19.1)	17.5 (16.3,18.6)	16.2 (15.1,17.3)	-26.2	< 0.01
65-79	13.0 (12.0,13.9)	11.9 (11.0,12.7)	11.9 (11.1,12.7)	11.1 (10.3,11.9)	10.9 (10.1,11.7)	10.8 (10.0,11.6)	10.4 (9.6,11.1)	9.8 (9.0,10.6)	9.8 (9.0,10.7)	-24.1	0.11
80+	5.6 (4.5,6.6)	5.7 (4.4,7.0)	5.6 (4.7,6.4)	4.9 (4.0,5.8)	4.8 (3.9,5.7)	4.4 (3.4,5.5)	4.2 (3.3,5.0)	4.1 (3.2,4.9)	4.3 (3.5,5.2)	-22.2	0.04
**Heavy drinker**											
Both sexes											
<18	6.4 (5.8,6.9)	7.2 (6.6,7.7)	3.4 (3.0,3.9)	1.3 (1.1,1.6)	1.2 (0.9,1.4)	1.5 (1.1,1.8)	1.1 (0.8,1.4)	1.0 (0.7,1.3)	0.4 (0.2,0.6)	-93.5	0.01
18-49	14.7 (14.3,15.1)	15.6 (15.2,16.1)	18.2 (17.7,18.6)	9.4 (9.0,9.7)	6.8 (6.5,7.2)	12.4 (11.8,12.9)	10.7 (10.2,11.2)	15.5 (14.9,16)	7.6 (7.1,8.1)	-48.2	< 0.01
50-64	13.7 (13,14.3)	14.3 (13.6,14.9)	15.7 (15.1,16.4)	8.8 (8.3,9.2)	6.9 (6.3,7.5)	12.1 (11.3,12.9)	11.3 (10.6,12)	16.8 (16.0,17.6)	8.6 (8.0,9.3)	-36.9	< 0.01
65-79	11.0 (10.3,11.7)	11.7 (11.0,12.4)	12.4 (11.7,13)	8.5 (7.9,9.1)	5.4 (4.9,5.8)	10.2 (9.5,10.8)	9.5 (8.9,10)	12.7 (12,13.4)	7.2 (6.6,7.8)	-34.2	< 0.01
80+	6.6 (5.6,7.6)	8.8 (7.7,10.0)	7.5 (6.6,8.4)	5.6 (4.8,6.3)	3.6 (3.0,4.2)	6.3 (5.4,7.1)	6.4 (5.5,7.3)	8.1 (7.1,9.1)	5.0 (4.1,5.8)	-24.6	0.01
Male											
<18	6.3 (5.6,7.0)	7.8 (7.0,8.5)	3.3 (2.7,3.9)	1.2 (0.8,1.5)	1.3 (1.0,1.7)	1.4 (0.9,1.8)	1.0 (0.6,1.4)	1.0 (0.5,1.5)	0.2 (0.0,0.4)	-97.0	0.02

18-49	16.8 (16.2,17.4)	17.9 (17.2,18.6)	21.1 (20.4,21.8)	10.8 (10.2,11.4)	8.1 (7.5,8.7)	13.1 (12.3,13.9)	11.5 (10.7,12.2)	17.1 (16.2,18.0)	8.0 (7.3,8.7)	-52.4	< 0.01
50-64	15.4 (14.5,16.4)	16.4 (15.4,17.4)	17.2 (16.2,18.2)	9.4 (8.7,10.2)	7.6 (6.7,8.6)	13.3 (12.0,14.6)	12.2 (11.2,13.2)	17.8 (16.5,19.0)	9.0 (7.9,10.0)	-42.0	< 0.01
65-79	12.0 (10.9,13.2)	12.6 (11.6,13.7)	14.2 (13.0,15.3)	8.8 (7.9,9.7)	5.2 (4.6,5.8)	10.6 (9.7,11.6)	9.9 (9.0,10.7)	13.2 (12.2,14.3)	7.5 (6.7,8.4)	-37.6	< 0.01
80+	5.1 (3.7,6.5)	9.0 (6.8,11.1)	6.9 (5.4,8.4)	6.1 (4.8,7.4)	3.5 (2.6,4.4)	5.6 (4.4,6.8)	5.0 (4.0,6.1)	8.4 (6.6,10.1)	4.5 (3.3,5.7)	-12.2	0.04
Female											
<18	6.5 (5.7,7.2)	6.6 (5.8,7.3)	3.6 (2.8,4.3)	1.5 (1.1,1.9)	1.0 (0.7,1.3)	1.5 (1.1,2.0)	1.2 (0.7,1.6)	1.1 (0.7,1.5)	0.6 (0.3,1.0)	-90.2	< 0.01
18-49	12.7 (12.1,13.2)	13.5 (12.9,14.1)	15.4 (14.8,16)	8.0 (7.5,8.4)	5.6 (5.2,6.1)	11.6 (10.9,12.4)	10.0 (9.3,10.7)	13.8 (13.1,14.6)	7.2 (6.6,7.8)	-42.9	< 0.01
50-64	12.0 (11.1,12.8)	12.2 (11.4,13.0)	14.3 (13.4,15.1)	8.1 (7.5,8.7)	6.2 (5.5,7.0)	11.0 (10.1,11.9)	10.5 (9.6,11.5)	15.9 (14.8,16.9)	8.3 (7.4,9.2)	-30.7	< 0.01
65-79	10.1 (9.1,11.0)	10.9 (10.0,11.8)	10.7 (9.9,11.6)	8.3 (7.5,9.0)	5.5 (4.9,6.1)	9.7 (8.8,10.7)	9.1 (8.3,9.8)	12.2 (11.3,13.1)	6.9 (6.2,7.7)	-31.0	< 0.01
80+	7.5 (6.2,8.9)	8.7 (7.4,10.1)	7.8 (6.7,8.9)	5.2 (4.3,6.1)	3.7 (2.9,4.5)	6.8 (5.6,8.0)	7.4 (6.0,8.7)	7.9 (6.7,9.1)	5.4 (4.2,6.6)	-28.9	< 0.01
**High blood cholesterol[a]**											
Both sexes											
<18	-	-	-	-	-	-	-	-	-	-	-
18-49	-	-	-	-	-	-	-	4.2 (3.9,4.6)	3.7 (3.4,4.1)	-11.4	< 0.01
50-64	-	-	-	-	-	-	-	19.6 (18.7,20.4)	18.7 (17.8,19.5)	-4.5	< 0.01
65-79	-	-	-	-	-	-	-	30.3 (29.2,31.3)	28.7 (27.7,29.6)	-5.4	< 0.01
80+	-	-	-	-	-	-	-	25.8 (24.1,27.6)	25.0 (23.4,26.7)	-3.1	< 0.01
Male											

<18	-	-	-	-	-	-	-	-	-	-	-
18-49	-	-	-	-	-	-	-	5.6 (5.1,6.2)	4.9 (4.4,5.4)	-13.5	< 0.01
50-64	-	-	-	-	-	-	-	22.4 (21.2,23.7)	21.9 (20.6,23.2)	-2.2	< 0.01
65-79	-	-	-	-	-	-	-	32.4 (30.9,34.0)	31.2 (29.8,32.6)	-3.9	< 0.01
80+	-	-	-	-	-	-	-	25.1 (22.5,27.6)	27.3 (24.6,30.1)	9.1	< 0.01
Female											
<18	-	-	-	-	-	-	-	-	-	-	-
18-49	-	-	-	-	-	-	-	2.8 (2.4,3.3)	2.6 (2.3,3.0)	-7.4	0.05
50-64	-	-	-	-	-	-	-	16.8 (15.6,17.9)	15.5 (14.5,16.6)	-7.3	< 0.01
65-79	-	-	-	-	-	-	-	28.3 (26.9,29.7)	26.3 (24.9,27.6)	-7.0	< 0.01
80+	-	-	-	-	-	-	-	26.4 (24.0,28.8)	23.2 (21.3,25.2)	-12.0	< 0.01

[a] Populations less than 18 years of age were excluded in the Canadian Community Health Survey for questions related to high blood cholesterol.

Supplemental Table S6: Age- and sex-standardized prevalence of risk factors, by provinces and territories in Canada, from 2001 to 2017/2018. Source: Canadian Community Health Survey (CCHS) - Public Use Micro Data. Data harmonized using *cchsflow*. The CCHS underwent a major survey redesign starting in 2015. Bootstrap weights were used for 2015 and 2017. Age and sex standardization were done using the 2011 Canadian Census population.

Risk factor; provinces and territories	Year, prevalence (%, 95% CI)									Relative change, %	p-value
	2001	2003	2005	2007	2009	2011	2013	2015	2017		
**Hypertension**											
Alberta	11.2 (10.5,12.0)	12.9 (12.1,13.8)	13.7 (12.8,14.6)	15.4 (14.4,16.4)	15.9 (14.8,17.0)	16.3 (15.2,17.5)	16.2 (15.1,17.3)	14.3 (13.4,15.1)	14.8 (14.0,15.7)	31.9	< 0.01
British Columbia	12.2 (11.6,12.8)	14.2 (13.5,14.9)	14.2 (13.4,15.0)	15.4 (14.6,16.2)	16.1 (15.2,17.0)	17.3 (16.3,18.4)	16.8 (15.7,17.8)	14.3 (13.5,15.1)	15.9 (15.1,16.7)	30.2	0.6
Manitoba	14.6 (13.5,15.7)	15.3 (14.0,16.6)	16.4 (15.1,17.6)	18.6 (17.2,20.1)	18.1 (16.5,19.7)	19.2 (17.4,20.9)	20.4 (18.7,22.0)	17.9 (16.4,19.4)	19.1 (17.5,20.7)	31.0	< 0.01
New Brunswick	15.5 (14.3,16.7)	17.5 (16.2,18.8)	20.5 (19.0,22.0)	20.6 (19.3,21.9)	23.1 (21.5,24.7)	24.0 (22.2,25.7)	25.1 (23.3,26.8)	21.0 (19.3,22.7)	22.5 (20.8,24.2)	45.2	< 0.01
Newfoundland and Labrador	16.6 (15.1,18.0)	18.0 (16.4,19.6)	20.4 (18.8,22.0)	21.9 (20.3,23.6)	24.2 (22.3,26.2)	23.9 (21.8,25.9)	25.2 (23.2,27.3)	22.7 (20.7,24.6)	22.6 (20.7,24.4)	36.1	< 0.01
Nova Scotia	17.4 (16.1,18.6)	19.5 (17.9,21.1)	19.2 (17.9,20.6)	21.1 (19.7,22.5)	22.5 (20.9,24.2)	24.2 (22.3,26.1)	21.8 (20.3,23.3)	20.4 (19.0,21.9)	20.9 (19.6,22.3)	20.6	< 0.01
Ontario	14.0 (13.6,14.5)	15.8 (15.3,16.4)	16.3 (15.8,16.9)	17.5 (17.0,18.1)	18.5 (17.9,19.1)	18.7 (18.0,19.4)	19.7 (19.0,20.4)	17.0 (16.4,17.7)	17.0 (16.3,17.7)	21.0	0.0
Prince Edward Island	15.3 (13.8,16.8)	16.5 (14.6,18.4)	17.6 (15.5,19.7)	19.3 (17.3,21.2)	19.2 (16.8,21.6)	21.7 (19.2,24.3)	24.4 (21.8,27.1)	18.6 (16.8,20.3)	18.7 (16.9,20.5)	22.4	< 0.01
Quebec	13.4 (12.8,14.0)	15.3 (14.6,16.0)	16.4 (15.7,17.0)	17.1 (16.4,17.9)	18.3 (17.5,19.1)	18.2 (17.4,19.1)	18.5 (17.7,19.3)	16.1 (15.4,16.8)	15.9 (15.3,16.5)	18.4	0.1
Saskatchewan	13.6 (12.7,14.5)	16.1 (15.1,17.2)	17.1 (16.0,18.1)	19.1 (17.8,20.3)	19.6 (18.2,21.0)	19.8 (18.3,21.2)	18.2 (16.9,19.5)	18.4 (16.9,19.8)	18.5 (16.9,20.0)	36.3	< 0.01
Yukon/Northwest Territories/Nunavut	8.4 (7.1,9.7)	10.4 (8.9,12.0)	9.6 (8.1,11.0)	11.7 (10.1,13.2)	12.5 (10.8,14.1)	12.5 (11.0,14.0)	13.9 (12.2,15.5)	14.1 (12.4,15.8)	13.2 (11.8,14.6)	57.5	0.0

**Diabetes**											
Alberta	3.6 (3.2,4.0)	3.8 (3.3,4.2)	4.2 (3.7,4.7)	4.9 (4.3,5.5)	5.5 (4.8,6.1)	5.7 (4.8,6.5)	5.4 (4.8,6.0)	5.0 (4.5,5.6)	6.2 (5.6,6.8)	70.7	< 0.01
British Columbia	4.1 (3.7,4.5)	4.9 (4.4,5.3)	4.7 (4.3,5.2)	5.3 (4.8,5.8)	5.7 (5.1,6.2)	5.7 (5.1,6.3)	5.9 (5.3,6.5)	5.7 (5.1,6.2)	5.6 (5.1,6.1)	37.2	0.5
Manitoba	4.3 (3.7,5.0)	5.5 (4.7,6.3)	4.7 (4.1,5.4)	5.3 (4.5,6.0)	6.1 (5.2,7.0)	6.9 (5.7,8.1)	6.7 (5.7,7.6)	6.0 (5.1,6.9)	6.6 (5.6,7.6)	53.0	0.1
New Brunswick	5.5 (4.7,6.3)	5.7 (4.9,6.5)	6.4 (5.5,7.2)	8.1 (7.2,9.0)	7.6 (6.6,8.5)	8.6 (7.4,9.8)	8.5 (7.5,9.6)	8.3 (7.1,9.5)	10.0 (8.8,11.3)	82.7	< 0.01
Newfoundland and Labrador	6.2 (5.3,7.2)	6.8 (5.7,7.9)	7.3 (6.3,8.4)	9.5 (8.3,10.6)	8.7 (7.5,9.8)	9.9 (8.5,11.4)	9.5 (8.3,10.7)	9.8 (8.5,11.1)	9.0 (7.8,10.2)	44.1	< 0.01
Nova Scotia	5.5 (4.8,6.2)	5.8 (5.0,6.6)	7.0 (6.2,7.8)	7.5 (6.6,8.4)	8.5 (7.5,9.6)	9.1 (8.0,10.3)	8.4 (7.4,9.3)	9.2 (8,10.4)	8.4 (7.4,9.5)	52.5	< 0.01
Ontario	4.5 (4.2,4.8)	4.9 (4.6,5.2)	5.0 (4.7,5.3)	6.4 (6.0,6.9)	7.3 (6.9,7.8)	7.0 (6.6,7.5)	7.4 (6.9,7.8)	7.0 (6.5,7.5)	7.2 (6.7,7.7)	60.7	0.0
Prince Edward Island	5.0 (4.1,5.9)	5.2 (4.1,6.4)	6.8 (5.5,8.1)	6.6 (5.4,7.8)	7.6 (5.8,9.4)	8.1 (6.4,9.8)	9.3 (7.5,11.1)	8.0 (6.6,9.3)	8.4 (7.0,9.7)	65.7	< 0.01
Quebec	4.4 (4.0,4.8)	4.8 (4.4,5.3)	5.4 (5.0,5.8)	6.1 (5.7,6.6)	6.0 (5.5,6.5)	6.6 (6.0,7.1)	7.2 (6.7,7.8)	6.5 (6.1,7.0)	6.5 (6.1,6.9)	48.8	0.1
Saskatchewan	4.3 (3.7,4.8)	5.0 (4.3,5.6)	5.4 (4.7,6.0)	6.4 (5.7,7.1)	6.9 (6.0,7.8)	6.6 (5.8,7.4)	7.0 (6.1,7.8)	6.7 (5.8,7.5)	6.5 (5.5,7.4)	52.5	0.0
Yukon/Northwest Territories/Nunavut	2.8 (2.0,3.5)	3.4 (2.5,4.3)	3.2 (2.4,4.0)	3.6 (2.8,4.4)	4.8 (3.6,5.9)	5.1 (3.9,6.2)	5.7 (4.6,6.8)	4.8 (3.8,5.9)	6.7 (5.3,8.0)	142.0	0.4
**Obesity**											
Alberta	14.0 (13.2,14.8)	14.3 (13.4,15.2)	14.6 (13.7,15.5)	16.6 (15.6,17.6)	16.7 (15.6,17.9)	16.9 (15.8,18.1)	18.6 (17.3,19.9)	18.5 (17.4,19.5)	19.2 (18.1,20.3)	37.0	< 0.01
British Columbia	11.0 (10.4,11.6)	10.8 (10.1,11.4)	12.6 (11.9,13.4)	11.3 (10.5,12.0)	12.2 (11.3,13.0)	13.1 (12.1,14.1)	14.1 (13.1,15.1)	13.4 (12.6,14.3)	13.9 (13.0,14.8)	26.3	< 0.01
Manitoba	15.6 (14.5,16.7)	16.3 (14.9,17.6)	17.1 (15.8,18.4)	16.8 (15.6,18.1)	20.3 (18.4,22.1)	18.1 (16.5,19.8)	21.2 (19.4,22.9)	19.8 (18.2,21.5)	19.7 (18.0,21.4)	26.4	0.1
New Brunswick	18.2 (16.9,19.4)	18.1 (16.6,19.6)	20.9 (19.3,22.4)	19.4 (18.0,20.7)	24.7 (22.8,26.5)	23.9 (22.0,25.7)	23.3 (21.6,25.1)	26.8 (24.7,28.9)	25.2 (23.1,27.4)	39.0	< 0.01

Newfoundland and Labrador	18.4 (16.9,19.8)	18.6 (17.0,20.2)	22.2 (20.5,24.0)	22.2 (20.5,24.0)	24.6 (22.6,26.6)	23.8 (21.6,26.1)	26.7 (24.4,28.9)	28.5 (26.1,30.9)	26.2 (24.0,28.4)	42.8	< 0.01
Nova Scotia	18.3 (17.0,19.6)	18.7 (17.1,20.4)	19.1 (17.7,20.5)	20.2 (18.7,21.7)	22.3 (20.4,24.2)	22.0 (20.1,23.9)	24.5 (22.6,26.3)	23.7 (21.9,25.4)	23.7 (21.9,25.4)	29.4	< 0.01
Ontario	13.5 (13.0,14.0)	13.9 (13.4,14.4)	14.5 (14.0,15.0)	14.9 (14.3,15.4)	15.9 (15.3,16.6)	16.3 (15.6,17.0)	17.0 (16.3,17.7)	17.1 (16.3,17.8)	16.6 (15.9,17.3)	23.1	0.3
Prince Edward Island	15.7 (14.1,17.2)	18.2 (15.9,20.4)	20.3 (17.9,22.6)	20.1 (17.8,22.3)	18.7 (16.1,21.3)	21.5 (18.7,24.3)	22.4 (19.7,25.1)	20.7 (18.5,23.0)	23.0 (20.6,25.3)	46.6	< 0.01
Quebec	11.6 (11.0,12.2)	13.1 (12.4,13.8)	13.3 (12.7,13.9)	13.8 (13.1,14.5)	14.9 (14.1,15.7)	15.4 (14.6,16.3)	16.6 (15.7,17.4)	15.9 (15.1,16.6)	17.5 (16.8,18.2)	50.9	0.1
Saskatchewan	16.5 (15.5,17.5)	18.1 (16.8,19.3)	18.9 (17.6,20.1)	20.3 (18.9,21.6)	19.1 (17.7,20.5)	20.8 (19.1,22.4)	21.9 (20.4,23.5)	22.0 (20.2,23.7)	23.4 (21.4,25.4)	42.4	< 0.01
Yukon/Northwest Territories/Nunavut	18.2 (16.4,20.0)	18.6 (16.6,20.7)	20.0 (18.0,22.1)	18.6 (16.8,20.4)	18.5 (16.6,20.4)	19.8 (17.9,21.7)	21.8 (19.8,23.8)	25.6 (23.3,27.9)	25.6 (23.2,27.9)	40.4	< 0.01
**Physical inactivity**											
Alberta	44.4 (43.2,45.6)	43.5 (42.2,44.9)	44.6 (43.3,46.0)	45.1 (43.7,46.5)	43.4 (41.9,45.0)	44.5 (42.8,46.3)	42.6 (40.9,44.4)	36.4 (35,37.7)	36.1 (34.8,37.4)	-18.6	< 0.01
British Columbia	38.0 (37.1,38.9)	38.9 (37.8,40.0)	40.3 (39.2,41.4)	40.2 (39.0,41.3)	39.7 (38.3,41.0)	39.1 (37.6,40.5)	36.1 (34.6,37.6)	35.4 (34.1,36.7)	35.6 (34.4,36.9)	-6.2	0.1
Manitoba	49.3 (47.7,50.9)	48.3 (46.3,50.2)	50.8 (49.0,52.6)	46.2 (44.3,48.1)	46.0 (43.7,48.3)	45.6 (43.2,48.0)	45.3 (43.1,47.4)	40.6 (38.5,42.7)	42.0 (39.8,44.2)	-14.8	0.1
New Brunswick	54.8 (53.1,56.4)	53.2 (51.3,55.2)	53.0 (51.2,54.8)	51.5 (49.7,53.3)	48.2 (46.2,50.2)	48.0 (45.8,50.2)	49.9 (47.8,52.0)	42.7 (40.3,45.1)	44.1 (41.8,46.3)	-19.5	< 0.01
Newfoundland and Labrador	56.8 (54.9,58.7)	54.1 (51.9,56.3)	53.9 (51.8,55.9)	53.4 (51.3,55.5)	51.4 (49.2,53.7)	48.9 (46.3,51.4)	50.4 (48.0,52.8)	45.2 (42.5,47.8)	45.4 (42.9,47.9)	-20.1	< 0.01
Nova Scotia	53.1 (51.5,54.8)	50.2 (48.1,52.3)	50.5 (48.6,52.4)	50.5 (48.6,52.4)	46.6 (44.5,48.8)	45.8 (43.5,48.1)	45.0 (42.9,47.0)	39.6 (37.7,41.6)	38.5 (36.6,40.3)	-27.6	0.0
Ontario	50.1 (49.4,50.8)	47.6 (46.8,48.3)	46.3 (45.5,47.0)	49.3 (48.5,50.2)	48.8 (47.9,49.6)	45.6 (44.6,46.5)	45.7 (44.7,46.6)	40.5 (39.5,41.5)	43.6 (42.6,44.6)	-12.9	0.1
Prince Edward Island	53.0 (50.7,55.2)	53.8 (50.9,56.6)	56.1 (53.3,59.0)	51.9 (49.2,54.6)	47.9 (44.7,51.0)	47.7 (44.3,51.1)	50.5 (47.3,53.6)	40.0 (37.3,42.6)	41.6 (39.0,44.2)	-21.5	< 0.01
Quebec	54.9 (54.0,55.8)	51.1 (50.1,52.1)	50.9 (50.0,51.8)	51.8 (50.8,52.9)	49.4 (48.3,50.5)	49.2 (48.1,50.4)	48.1 (46.9,49.2)	42.4 (41.4,43.4)	45.1 (44.1,46.1)	-17.8	< 0.01

Saskatchewan	49.5 (48.1,50.9)	48.6 (46.9,50.2)	49.1 (47.5,50.7)	51.2 (49.6,52.8)	48.3 (46.5,50.2)	46.6 (44.5,48.7)	46.5 (44.6,48.5)	40.1 (37.9,42.3)	39.7 (37.5,41.8)	-19.8	0.0
Yukon/Northwest Territories/Nunavut	42.8 (40.5,45.0)	45.4 (42.8,48.0)	45.8 (43.3,48.3)	50.6 (48.2,52.9)	48.8 (46.4,51.2)	44.7 (42.3,47.0)	43.9 (41.5,46.2)	30.0 (27.4,32.5)	29.2 (26.9,31.6)	-31.7	1.0
**Current smoker**											
Alberta	27.2 (26.2,28.3)	22.7 (21.6,23.9)	22.5 (21.4,23.7)	22.0 (20.9,23.1)	22.6 (21.2,24.0)	21.4 (19.9,22.8)	19.4 (18.2,20.7)	17.4 (16.4,18.4)	15.8 (14.8,16.7)	-42.1	< 0.01
British Columbia	20.2 (19.5,20.9)	18.4 (17.5,19.2)	17.9 (17.1,18.8)	17.9 (17.0,18.9)	16.4 (15.5,17.4)	15.0 (14.0,16.0)	15.0 (13.9,16.0)	13.5 (12.5,14.4)	12.0 (11.2,12.9)	-40.5	0.0
Manitoba	24.3 (22.9,25.6)	22.3 (20.7,23.9)	20.3 (18.8,21.7)	22.6 (21.0,24.1)	19.4 (17.6,21.1)	19.4 (17.4,21.3)	17.4 (15.7,19.2)	16.4 (14.7,18.0)	14.5 (12.8,16.2)	-40.3	0.4
New Brunswick	25.6 (24.2,27.0)	24.7 (23.0,26.4)	22.2 (20.7,23.7)	22.7 (21.2,24.2)	21.7 (19.9,23.4)	22.0 (20.2,23.9)	20.8 (19.1,22.5)	17.7 (15.9,19.6)	14.3 (12.7,15.9)	-44.1	1.0
Newfoundland and Labrador	28.4 (26.6,30.1)	23.7 (21.8,25.5)	22.7 (21.0,24.4)	24.3 (22.5,26.1)	22.7 (20.7,24.7)	22.7 (20.5,24.9)	20.1 (18.3,22.0)	21.5 (19.2,23.8)	20.4 (18.2,22.7)	-27.9	0.4
Nova Scotia	27.7 (26.3,29.2)	22.8 (21.0,24.7)	22.2 (20.7,23.7)	23.8 (22.1,25.4)	22.6 (20.7,24.4)	22.6 (20.5,24.6)	21.2 (19.4,23.0)	18.6 (16.9,20.2)	17.6 (16.2,19.1)	-36.4	0.6
Ontario	24.0 (23.4,24.6)	22.0 (21.4,22.6)	21.0 (20.4,21.6)	19.9 (19.2,20.5)	18.6 (17.9,19.2)	18.9 (18.2,19.7)	17.4 (16.7,18.1)	16.1 (15.3,16.9)	14.9 (14.2,15.7)	-37.8	0.3
Prince Edward Island	27.2 (25.3,29.2)	23.3 (20.9,25.8)	22.0 (19.6,24.5)	21.0 (18.7,23.2)	21.3 (18.5,24.1)	21.0 (18.1,23.8)	19.2 (16.7,21.6)	15.3 (13.3,17.3)	16.4 (14.4,18.5)	-39.7	0.8
Quebec	29.0 (28.1,29.8)	25.6 (24.7,26.5)	24.3 (23.5,25.0)	23.8 (23.0,24.7)	22.5 (21.5,23.4)	22.2 (21.2,23.2)	20.0 (19.1,20.9)	18.0 (17.3,18.8)	17.7 (17.0,18.5)	-38.8	0.5
Saskatchewan	26.9 (25.7,28.2)	23.3 (21.9,24.7)	23.6 (22.3,25.0)	25.0 (23.5,26.4)	21.9 (20.4,23.5)	21.4 (19.8,23.0)	21.1 (19.5,22.7)	18.6 (16.9,20.2)	17.7 (15.9,19.5)	-34.4	0.6
Yukon/Northwest Territories/Nunavut	44.3 (42.0,46.5)	38.7 (36.1,41.2)	37.4 (34.9,39.8)	37.9 (35.7,40.2)	39.6 (37.2,42.0)	37.3 (34.9,39.6)	38.1 (35.8,40.4)	19.7 (17.5,21.9)	18.0 (16.0,20.0)	-59.4	< 0.01
**Heavy drinker[a]**											
Alberta	12.5 (11.7,13.2)	13.1 (12.2,14.0)	13.8 (12.9,14.8)	-	-	-	-	13.0 (12.0,13.9)	-	-	< 0.01
British Columbia	13.6 (13.0,14.2)	13.5 (12.7,14.2)	15.7 (15.0,16.5)	14.8 (13.9,15.6)	-	-	-	13.8 (12.9,14.7)	14.4 (13.5,15.3)	6.3	0.2

Manitoba	11.2 (10.2,12.2)	12.1 (10.8,13.3)	12.1 (10.9,13.3)	-	-	14.3 (12.7,16.0)	13.0 (11.5,14.6)	12.1 (10.6,13.5)	-	-	0.5
New Brunswick	9.4 (8.4,10.3)	10.9 (9.7,12.2)	11.6 (10.5,12.8)	-	-	-	-	11.6 (9.9,13.3)	-	-	0.0
Newfoundland and Labrador	12.5 (11.2,13.8)	13.4 (11.8,15.1)	13.3 (11.9,14.7)	15.1 (13.5,16.8)	15.6 (13.8,17.3)	17.4 (15.2,19.6)	-	14.4 (12.5,16.2)	-	-	0.1
Nova Scotia	11.1 (10.0,12.2)	12.1 (10.6,13.6)	12.6 (11.3,13.8)	14.4 (12.9,15.8)	-	-	-	13.4 (11.9,14.9)	-	-	0.7
Ontario	12.7 (12.3,13.2)	14.0 (13.5,14.5)	15.9 (15.3,16.4)	14.7 (14.1,15.2)	14.0 (13.4,14.6)	14.7 (14.0,15.4)	13.3 (12.7,13.9)	13.3 (12.7,14.0)	13.4 (12.7,14.1)	5.4	0.3
Prince Edward Island	8.9 (7.6,10.1)	9.7 (7.8,11.5)	11.2 (9.2,13.2)	-	-	-	11.7 (9.5,13.8)	14.4 (12.1,16.7)	11.5 (9.6,13.3)	29.6	0.0
Quebec	13.5 (12.8,14.1)	14.3 (13.6,15.0)	16.0 (15.3,16.6)	-	-	17.2 (16.3,18.0)	16.3 (15.5,17.2)	16.6 (15.8,17.3)	-	-	0.0
Saskatchewan	10.9 (10.0,11.8)	11.8 (10.7,13.0)	12.1 (11.1,13.1)	-	12.7 (11.4,14.1)	14.1 (12.7,15.6)	13.5 (12.1,14.9)	12.6 (11.1,14.2)	-	-	0.5
Yukon/Northwest Territories/Nunavut	13.6 (12.0,15.2)	15.9 (14.0,17.7)	15.2 (13.5,16.9)	-	-	-	-	19.6 (17.2,21.9)	-	-	< 0.01
**High blood cholesterol**											
Alberta	-	-	-	-	-	-	-	9.5 (8.7,10.2)	9.7 (8.9,10.5)	1.9	< 0.01
British Columbia	-	-	-	-	-	-	-	10.2 (9.4,11.0)	10.4 (9.7,11.1)	1.7	0.3
Manitoba	-	-	-	-	-	-	-	12.6 (11.4,13.9)	11.8 (10.5,13.0)	-6.5	< 0.01
New Brunswick	-	-	-	-	-	-	-	15.1 (13.6,16.7)	14.0 (12.7,15.4)	-7.3	< 0.01
Newfoundland and Labrador	-	-	-	-	-	-	-	17.8 (16.1,19.6)	17.8 (16.2,19.4)	-0.1	< 0.01
Nova Scotia	-	-	-	-	-	-	-	14.7 (13.4,15.9)	13.5 (12.3,14.6)	-8.3	< 0.01
Ontario	-	-	-	-	-	-	-	12.8 (12.2,13.4)	11.9 (11.4,12.5)	-6.9	< 0.01

Prince Edward Island	-	-	-	-	-	-	-	12.4 (10.9,13.9)	10.3 (8.9,11.6)	-17.3	0.0
Quebec	-	-	-	-	-	-	-	11.1 (10.5,11.7)	10.7 (10.2,11.2)	-3.7	0.1
Saskatchewan	-	-	-	-	-	-	-	11.4 (10.2,12.7)	10.4 (9.2,11.6)	-9.0	0.1
Yukon/Northwest Territories/Nunavut	-	-	-	-	-	-	-	8.2 (6.8,9.5)	7.7 (6.5,8.9)	-6.1	0.0

[a] Alcohol-related modules in the Canadian Community Health Survey were only asked for certain provinces and territories for 2007 onwards.
